# Harnessing engineered extracellular vesicles for enhanced therapeutic efficacy: advancements in cancer immunotherapy

**DOI:** 10.1186/s13046-025-03403-w

**Published:** 2025-05-02

**Authors:** Zheng Gong, Cheng Cheng, Chaonan Sun, Xiaoli Cheng

**Affiliations:** 1 Department of Radiology, Shengjing Hospital of China Medical University, Liaoning Province, Shenyang, 110004 China; 2 Department of Cardiology, Shengjing Hospital of China Medical University, Liaoning Province, Shenyang, 110004 China; 3 Department of Radiation Oncology, Cancer Hospital of China Medical University, Liaoning Cancer Hospital & Institute, No. 44 Xiaoheyan Road, Dadong District, Shenyang, Liaoning 110042 China

**Keywords:** Extracellular Vesicles (EVs), Engineered EVs, Cancer immunotherapy, Drug delivery, Therapeutics

## Abstract

Extracellular vesicles (EVs), particularly engineered variants, have emerged as promising tools in cancer immunotherapy due to their inherent ability to modulate immune responses and deliver therapeutic agents with high specificity and minimal toxicity. These nanometer-sized vesicles, which include exosomes (Exos) and other subtypes, naturally participate in intercellular communication and are capable of carrying a diverse range of bioactive molecules, including proteins, lipids, RNAs, and metabolites. Recent advancements in the biogenesis of engineered EVs, such as strategies to modify their surface characteristics and cargo, have significantly expanded their potential as effective vehicles for targeted cancer therapies. Tailoring the contents of EVs, such as incorporating immunomodulatory molecules or gene-editing tools (GETs), has shown promising outcomes in enhancing anti-tumor immunity and overcoming the immunosuppressive tumor microenvironment (TME). Moreover, optimizing delivery mechanisms, through both passive and active targeting strategies, is crucial for improving the clinical efficacy of EV-based therapies. This review provides an overview of recent developments in the engineering of EVs for cancer immunotherapy, focusing on their biogenesis, methods of content customization, and innovations in cargo delivery. Additionally, the review addresses the challenges associated with the clinical translation of EV-based therapies, such as issues related to scalability, safety, and targeted delivery. By offering insights into the current state of the field and identifying key areas for future research, this review aims to advance the application of engineered EVs in cancer treatment.

## Introduction

Cancer continues to represent a major global health challenge and remains one of the leading causes of death, with its incidence steadily increasing worldwide. According to the 2025 Global Cancer Statistics, solid tumors account for approximately 90% of all cancer diagnoses but comprise only 40% of clinical trials, most of which are still in the early stages of development [1, 2]. This discrepancy underscores the significant challenges in effectively treating solid tumors with existing therapeutic strategies. While traditional treatments such as surgery, chemotherapy, and radiotherapy are essential components of cancer management, they possess inherent limitations. Despite advancements in surgical techniques, tumor recurrence and metastasis remain critical issues, further exacerbated by the immunosuppressive TME, which impedes robust immune responses and efficient drug delivery [3–5].

To address these limitations, immunotherapy has emerged as a promising alternative, harnessing the body’s immune system to detect and eliminate cancer cells. However, challenges such as tumor immune evasion (TIE) and the systemic toxicity of treatments continue to impede the widespread success of immunotherapeutic strategies [6–8]. In this regard, EVs-nano-sized, membrane-bound particles secreted by cells-have attracted significant attention for their role in intercellular communication and their potential as vehicles for drug delivery [9, 10]. Among them, Exos (30–150 nm in diameter) have demonstrated promise in targeting cancer cells, enhancing immune responses, and delivering therapeutic agents with minimal toxicity [11, 12].

Engineered EVs offer a promising solution to the significant challenges faced in cancer immunotherapy. By modifying their formation, surface properties, and therapeutic contents, these vesicles can be optimized for improved targeting, enhanced immune modulation, and precise delivery of treatments such as cytokines, checkpoint inhibitors (CIs), and gene-editing tools (GETs) like CRISPR/Cas9 [13–16]. The intrinsic ability of Exos to traverse biological barriers, coupled with their biocompatibility, positions them as ideal candidates for targeted cancer therapy. Moreover, recent advancements in engineering techniques have further enhanced their potential, enabling controlled drug release and prolonged circulation times [17, 18]. Despite promising preclinical results, the clinical application of engineered EVs faces several challenges, particularly in large-scale production, purification, and standardization. Variability in the composition of natural EVs, along with the heterogeneous nature of tumors, complicates their clinical development [19, 20]. Additionally, safety concerns regarding off-target effects (OTE) and long-term stability must be addressed before engineered EVs can be widely implemented in clinical practice.

This review examines recent advancements in engineered EVs as a platform for cancer immunotherapy. We explore various strategies employed to modify EV properties, including cargo loading and surface functionalization, and assess their potential to enhance the efficacy and specificity of cancer treatments. Furthermore, we address the challenges that impede the clinical application of engineered EVs and propose innovative solutions to improve their scalability, safety, and therapeutic effectiveness. By providing a comprehensive overview of the current state-of-the-art in EV-based cancer therapies, this review aims to underscore the potential of engineered EVs as a transformative tool in the fight against cancer.

### The biogenesis and compositions of EVs

**Fig. 1:**
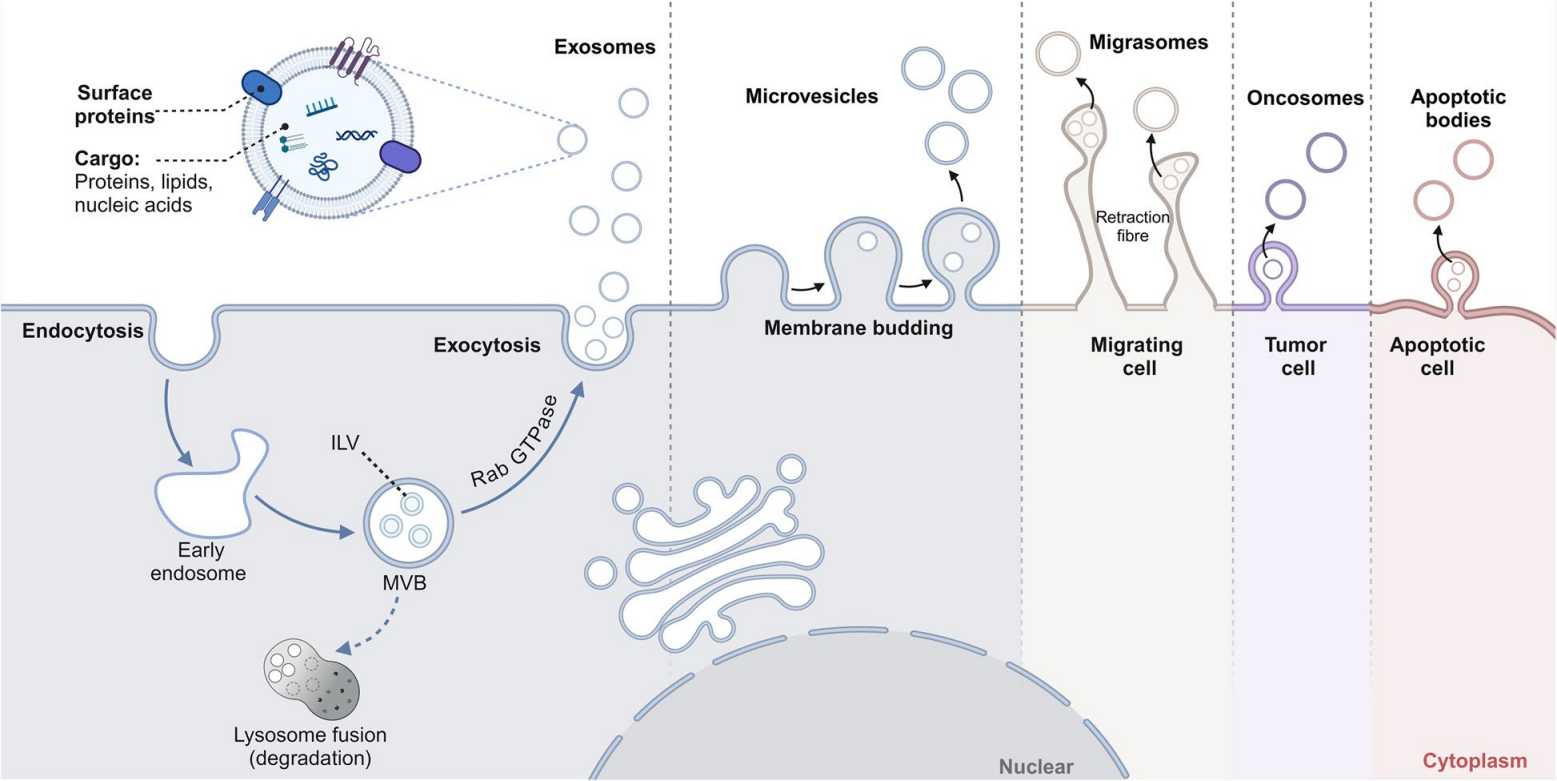
The biogenesis of extracellular vesicles (EVs). Exosomes are derived via the MVB/ILV pathway, whereas the microvesicles are produced by directly membrane budding. Migrasomes, oncosomes, and apoptotic bodies are particularly secreted by the migrating cell, tumor cell, and apoptotic cell, respectively. Created with BioRender.com

EVs are small, lipid bilayer-enclosed structures secreted by nearly all cell types, including malignant cells [21, 22]. These vesicles play a crucial role in intercellular communication by transporting a diverse array of biomolecules, such as proteins, lipids, RNA, and DNA. The biogenesis of EVs is a highly intricate and tightly regulated process (Fig. 1), involving distinct pathways that give rise to various vesicle subtypes, each possessing unique properties and functional implications in cancer immunotherapy [23, 24].

EVs are generally classified into three main subtypes based on their size and origin: exosomes (Exos), microvesicles (MVs), also known as ectosomes (Ectos), and apoptotic bodies (ABs). Exos, the most extensively studied subtype, range from 30 to 150 nm in diameter and originate from the endosomal system of cells [25, 26]. Their formation begins with the inward budding of the plasma membrane, leading to the generation of early endosomes. These early endosomes mature into late endosomes, which further invaginate to form multivesicular bodies (MVBs) containing intraluminal vesicles (ILVs). MVBs then fuse with the plasma membrane, releasing Exos into the extracellular space. Importantly, the cargo within Exos mirrors the molecular composition of their parent cells, which is influenced by processes such as signal transduction, cellular stress, and pathological conditions, including cancer [27, 28].

MVs (also referred to as Ectos) are larger than Exos, typically ranging from 100 nm to 1 µm in size. These vesicles are formed through the direct outward budding of the plasma membrane, followed by membrane scission, which releases them into the extracellular space. MVs encapsulate cargo derived from the cytoplasm, plasma membrane, and, in some cases, the nucleus. In the context of cancer, Ectos are particularly significant as they can transport tumor-associated antigens (TAAs), oncogenic proteins, and genetic material, thereby promoting tumor progression, metastasis, and immune evasion [25, 29].

ABs, the largest class of EVs, are generated during programmed cell death, or apoptosis. These vesicles range in size from 500 nm to several micrometers and contain fragmented cellular components, including organelles and chromatin. Although ABs are not typically involved in regular cellular communication, they play a role in the immune response to apoptotic cells, particularly by modulating the immune environment within tumors [21, 30].

The biogenesis of EVs is governed by a highly regulated process involving numerous key molecules essential for vesicle formation, cargo sorting, and secretion. In the case of Exos, proteins such as Alix, TSG101, and syntenin are critical for the formation of MVBs and the sorting of cargo into ILVs [22, 31]. The selective incorporation of specific molecules into EVs is an active and highly coordinated process. For example, proteins involved in antigen processing and presentation, such as major histocompatibility complex (MHC) molecules, are selectively enriched in Exos, allowing them to modulate immune responses. Furthermore, the cargo of EVs includes not only proteins but also lipids, mRNA, MicroRNA (miRNA), and DNA. The molecular content of EVs reflects the physiological state of the parent cell, a feature particularly relevant in cancer. Tumor-derived extracellular vesicles TEX can transport tumor-specific antigens, genetic mutations, and immunosuppressive factors that influence theTME and the immune system [32–34].

The cargo of EVs exhibits considerable diversity, reflecting the physiological state of the parent cell. This cargo includes lipids, proteins, and nucleic acids, each of which plays a distinct role in cellular signaling and disease processes. Exos, a specific subtype of EVs, are particularly enriched in lipids such as cholesterol, fatty acids, and eicosanoids, along with enzymes involved in lipid metabolism [35, 36]. These lipids regulate essential processes, including lipid trafficking, nuclear transcription, apoptosis, and inflammatory responses. Disruptions in exosomal lipid metabolism have been implicated in diseases such as atherosclerosis, cancer, and Alzheimer’s disease [37–39].

Proteins in Exos serve as valuable disease biomarkers due to their stability and detectability in small quantities. Notable exosomal proteins, such as the epidermal growth factor receptor (EGFR) and epithelial cell adhesion molecule (EpCAM), play significant roles in cancer progression through mechanisms including angiogenesis, proliferation, metastasis, and treatment resistance [40–43]. Proteomic analyses have revealed alterations in the exosomal proteome of cancer cells, including those from glioblastoma (GBM), where oncogenic proteins like EGFR variant III enhance invasiveness. The sorting of proteins into Exos involves mechanisms such as interaction with Lamp2 A, which targets specific motifs like KFERQ for inclusion [44, 45].

EVs also contain a variety of nucleic acids, including DNA and RNA, which facilitate communication between viral and host cells [46, 47]. They are capable of transporting viral, bacterial, and parasitic DNA and RNA, as observed in Plasmodium falciparum infections, where Exos aid in parasite dissemination [48, 49]. Notably, EVs lack double-stranded DNA and histones, indicating the absence of nuclear genomic DNA. The RNA content of Exos includes ribosomal RNA (rRNA), circular RNA (circRNA), miRNA, and long non-coding RNA (lncRNA) [9, 50]. These miRNAs play a critical role in regulating gene expression in recipient cells, and their sorting into Exos is governed by specific motifs, such as EXO-motifs, which are recognized by RNA-binding proteins like hnRNPA2B1 and SYNCRIP [51–56].

### The isolation methods of EVs

The isolation of EVs is crucial for both fundamental research and clinical applications, particularly in cancer immunotherapy [57]. A variety of methodologies have been developed for this purpose, each with distinct advantages and limitations depending on the EV type, biological source, and required purity [58]. Ultracentrifugation remains the most widely used technique, leveraging centrifugal force to separate EVs based on differences in size and density [59]. This method can be refined through differential centrifugation to remove cellular debris and density gradient ultracentrifugation to obtain more homogeneous EV populations. However, ultracentrifugation has several drawbacks, including long processing times, potential damage to EV structural integrity, and contamination risks from soluble proteins or non-EV particulates. Additionally, it requires specialized equipment and is highly operator-dependent, which can affect reproducibility [60].

To address the limitations of ultracentrifugation, alternative techniques that exploit the physical characteristics of EVs, such as size and morphology, have gained attention as supplementary methods [61]. Ultrafiltration uses semipermeable membranes to separate EVs from smaller particles, providing a more efficient alternative to ultracentrifugation. Size-exclusion chromatography (SEC) is another widely used technique that isolates EVs based on size, offering higher purity with minimal structural disruption [62]. SEC is particularly effective for isolating exosomes and is well-suited for downstream analytical processes such as proteomic and transcriptomic profiling. Polymer-based precipitation methods, including polyethylene glycol (PEG) precipitation, are also commonly employed due to their simplicity, cost-effectiveness, and scalability [63]. This approach involves adding a polymer to induce EV aggregation, which is then pelleted through low-speed centrifugation. While less time-consuming than ultracentrifugation, polymer precipitation may result in lower purity and the co-isolation of contaminant proteins or lipoproteins [64].

Emerging technologies, such as microfluidic-based isolation and immunoaffinity capture methods, hold promise for more efficient and selective EV isolation. Microfluidics enables the manipulation of small fluid volumes, allowing for high-throughput, label-free separation of EVs [65]. Immunoaffinity capture techniques, such as those targeting specific EV surface markers like CD63 or CD81, allow for the selective isolation of EV subpopulations [64]. These methods offer improved specificity, and are particularly advantageous for studies focused on disease-specific EV characterization.

Emerging methods like immunocapture and charge-based precipitation provide promising alternatives to traditional EV isolation techniques. These new methods offer higher sensitivity and specificity, making them ideal for isolating specific EV subpopulations, such as those with tumor-associated antigens (TAAs) or other disease biomarkers. Immunocapture techniques use antibodies targeting specific EV surface markers to selectively capture EVs from complex biological samples [66, 67]. This targeted isolation is crucial for cancer immunotherapy, enabling the extraction of EVs involved in tumor progression or immune modulation. These techniques also have straightforward workflows and potential for high-throughput applications, beneficial for personalized cancer therapies. Charge-based precipitation methods use surface charge differences to isolate EVs from other particles [68–70]. They are cost-effective, easy to use, and scalable, making them suitable for large-scale clinical applications. These methods can isolate a wide range of EVs, including exosomes, microvesicles, and apoptotic bodies, with minimal structural loss. They are adaptable for both research and clinical settings, providing flexible EV isolation protocols.

However, both methods have limitations. Immunocapture techniques may struggle with antibody specificity, leading to incomplete isolation of certain subpopulations. Variability in surface marker expression between patient samples or tumor types may affect isolation consistency and reproducibility. Charge-based precipitation methods, while isolating a broad range of EVs, may not effectively distinguish between EV subtypes, potentially co-isolating contaminants like soluble proteins or lipoproteins. The lack of precise size control in isolated EVs could also limit downstream analyses requiring high-purity samples. Despite these challenges, immunocapture and charge-based precipitation techniques hold promise for cancer immunotherapy. They enable precise isolation of EVs with therapeutic potential, facilitating targeted EV-based therapies. For example, EVs loaded with tumor-specific antigens or immune-stimulatory molecules could be used in cancer vaccination or immune modulation strategies. Their ability to isolate EVs from various biological fluids, such as blood, urine, or cerebrospinal fluid, opens new possibilities for non-invasive diagnostics and monitoring of cancer progression and treatment efficacy. Further optimization is needed to address limitations and ensure reproducibility, especially in clinical settings where standardization is critical for therapeutic success.

### Engineering strategies for EVs

#### Genetic engineering

Genetic modification of EVs has emerged as a powerful strategy to enhance their cargo and therapeutic potential, particularly in cancer immunotherapy. By leveraging recombinant DNA technology (rDNA), researchers can engineer parental cells to generate EVs enriched with specific proteins, peptides, or nucleic acids, which can be encapsulated within the vesicle or displayed on its surface. This approach harnesses the host cell’s natural machinery for the production and packaging of therapeutic agents, reducing the reliance on external loading methods while preserving EV structural integrity [71–73].

A common approach for genetic engineering involves the use of viral or non-viral vectors to introduce genes encoding therapeutic biomolecules into host cells. Viral vectors, such as adenoviruses, lentiviruses, and retroviruses, are frequently employed due to their high efficiency in delivering genetic material to target cells. These vectors are particularly beneficial for EV engineering, as they utilize the host cell’s machinery to ensure stable genetic incorporation without disrupting cellular function or triggering immune responses. For instance, lentiviral vectors enable stable transduction of parental cells, resulting in the continuous production of engineered EVs that carry therapeutic molecules, including cytokines, Small interfering RNAs (siRNAs), or tumor-targeting peptides [74].

A key area of focus in genetic engineering is the modification of exosomal surface proteins to enhance targeting specificity. Lysosomal-associated membrane protein 2B (LAMP-2B), a transmembrane protein located on exosomal membranes, is frequently used for its ability to present targeting ligands [75, 76]. By fusing target peptides to the N-terminal extracellular domain of LAMP-2B, researchers can engineer Exos to selectively bind to specific cell types, such as cancer cells, thereby improving the targeted delivery of therapeutic cargo [77]. Other surface proteins, including tetraspanins (CD9, CD63, CD81) and receptor proteins like Platelet-derived growth factor receptor (PDGFR), are also employed for surface modification, allowing for precise targeting applications.

Genetically engineering EVs offers several significant advantages over traditional exogenous loading methods, such as incubation or transfection. This approach allows for the production of large quantities of EVs with high loading efficiency, particularly for large and complex biomolecules like proteins and nucleic acids [78, 79]. Moreover, genetic modification preserves the natural characteristics of the EV membrane, ensuring that engineered EVs retain key functional properties, such as intercellular communication and membrane integrity, which are crucial for their therapeutic efficacy. Studies have demonstrated that EVs produced through genetic engineering do not exhibit significant changes in their physicochemical properties, such as size or surface markers, compared to their unmodified counterparts [80, 81].

Despite these advantages, several challenges persist in the genetic engineering of EVs. One major limitation is the potential overexpression of cytotoxic proteins, which could compromise host cell viability or trigger undesirable immune responses [82]. Additionally, the genetic modification process can be labor-intensive and may require substantial infrastructure, including the use of viral vectors and advanced cell culture techniques [83]. Furthermore, the stability of engineered EVs, especially under clinical storage and delivery conditions, remains an area that requires further optimization [72].

#### Surface modification

**Fig. 2:**
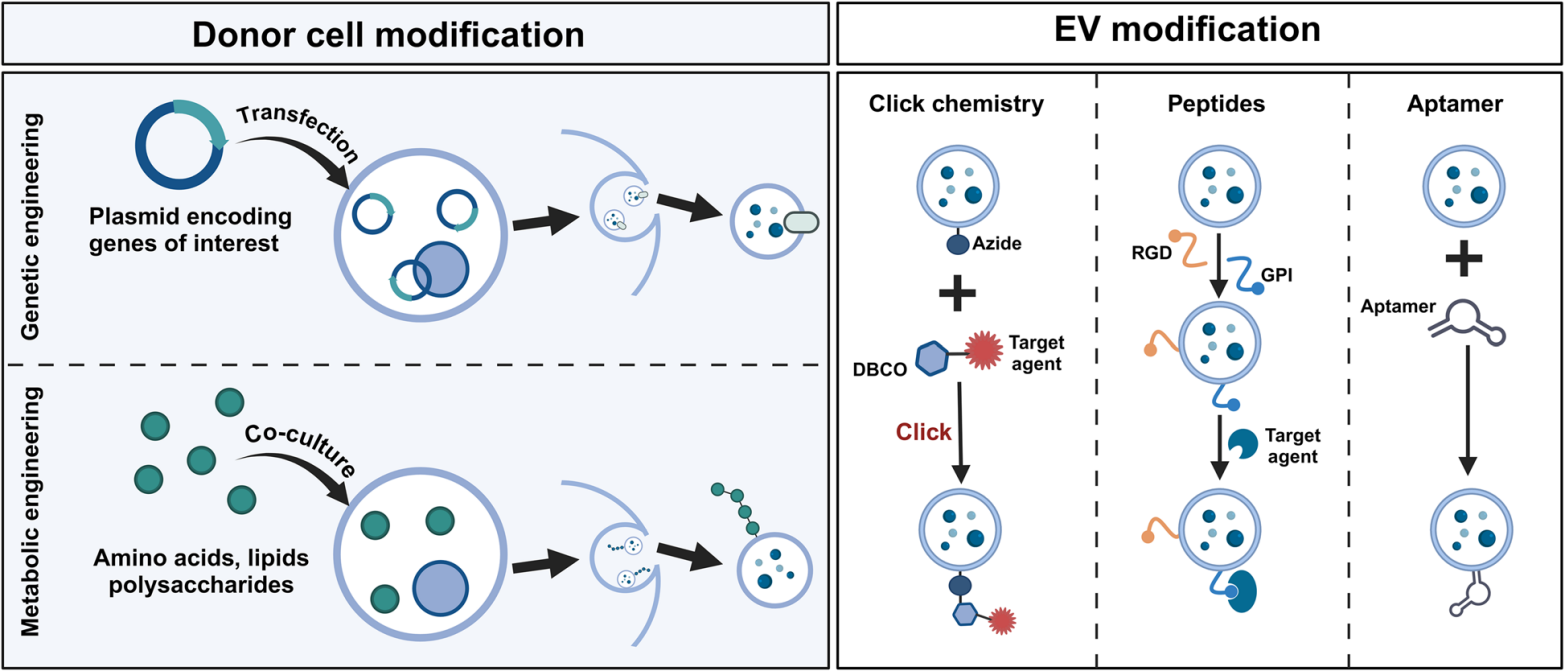
Currently utilized approaches for EV surface modification. Genetic and metabolic engineering methods are commonly employed to donor cell, thus indirectly modifying the surface of secreted EVs. For the direct modification of EV surface, click chemistry based on DBCO-Azide reaction, peptide (e.g. RGD and GPI) interaction, and aptamer attachment are the primary approaches. Created with BioRender.com

Currently, multiple strategies like the genetic engineering, covalent (e.g. click chemistry, metabolic engineering) and non-covalent (e.g. peptide, aptamer) modification for EV surface modification have been employed to optimize tumor targeting by introducing new functionalities through the incorporation of targeted molecules (Fig. 2).

Chemical strategies for surface modification primarily involve the covalent or non-covalent conjugation of targeting moieties to EVs. Among the most effective methods, copper-catalyzed azide-alkyne cycloaddition (CuAAC) has become a powerful technique for conjugating small molecules, peptides, and even larger biologics to the EV surface through bio-orthogonal reactions. This “click chemistry” approach offers exceptional specificity and minimal cytotoxicity, making it ideal for targeted DD applications [84]. Moreover, recent advancements in copper-free click chemistry and metabolic glycoengineering have further improved the clinical applicability of this method, ensuring that EVs retain their functionality and are non-toxic for in vivo use [85].

Alternatively, non-covalent approaches, such as electrostatic interactions, receptor-ligand binding, and hydrophobic insertion, can also be employed. These methods are generally simpler and less technically demanding than covalent techniques, allowing for the attachment of targeting peptides or small molecules without compromising the structural integrity of the EV. For example, the incorporation of cationic lipids onto the EV surface can introduce a positive charge, enhancing the vesicle’s interaction with negatively charged tumor cells and improving its uptake [86].

In the field of biological modifications, EVs are often engineered by attaching targeting peptides, aptamers, or antibodies to their surface to enhance specificity for cancer cells. Targeting peptides such as c(RGDyK) have been widely used to improve the homing ability of EVs to tumor cells by binding to integrin receptors (e.g., αvβ3) that are overexpressed on tumor vasculature or cells within theTME [87]. Similarly, peptides like TLyp-1 and c-Met-targeting peptides have been employed to direct EVs specifically to cancerous tissues [88, 89].

Aptamers, short single-stranded oligonucleotides with high affinity for specific targets, have also been explored for their potential to guide EVs toward cancer cells with high specificity. For instance, E3-aptamer-modified EVs carrying siRNA targeting SIRT6 have demonstrated promising antitumor effects in prostate cancer models [90]. Additionally, aptamer-coated EVs have shown effective targeting in cancers such as hepatocellular carcinoma (HCC) by binding to EpCAM [91]. These biological modifications enable highly specific targeting and hold significant promise in precision medicine, where individualized therapeutic approaches are essential.

Moreover, the modification of EVs with immunological molecules, such as antibodies or immune CIs, has been explored to enhance the immune response against tumors. For example, Exos engineered with anti-CD3 and anti-HER2 antibodies have been shown to promote T-cell activation and boost immune responses in breast cancer [92]. Similarly, incorporating immune CIs like Programmed cell death protein 1 (PD-1) on the surface of EVs has been found to improve immune evasion and enhance tumor targeting, opening new avenues for novel immunotherapies [93].

#### Internal payload

The ability to efficiently load therapeutic agents into EVs, particularly TEXs, is critical to their effectiveness as DD vehicles in cancer immunotherapy [94]. Several strategies have been developed to incorporate a wide range of cargo, including small molecule drugs, nucleic acids, proteins, and peptides, into the internal lumen of EVs. These approaches aim to optimize the therapeutic potential of EVs while minimizing OTE and maximizing therapeutic efficacy. In this section, we will review the engineering strategies used to load internal payloads into EVs, focusing on optimizing loading methods, addressing challenges associated with different types of cargo, and improving overall delivery efficiency.

The loading of therapeutic agents into EVs can be achieved through various techniques, each tailored to specific types of cargo. Traditional methods such as incubation, electroporation, sonication, and freeze–thaw cycles are commonly used to load small molecule drugs and nucleic acids. For instance, the co-incubation method is effective for loading hydrophobic compounds, which can easily diffuse into the lipid bilayer of EVs. In contrast, hydrophilic drugs typically require more advanced techniques to overcome the permeability barriers of the lipid bilayer [95].

Electroporation and sonication are commonly used to load larger or more complex molecules, such as therapeutic RNAs or proteins, into EVs. However, these methods have their limitations. Electroporation, while effective in improving loading efficiency, can cause membrane damage, potentially compromising EV integrity or leading to RNA aggregation [96, 97]. Similarly, sonication, although efficient for drug loading, can alter the EV structure, negatively affecting its stability and function. These drawbacks underscore the need for further optimization of EV loading techniques to improve both efficiency and safety.

To address the limitations of conventional methods, more advanced and sophisticated techniques have been developed. For example, extrusion and freeze–thaw cycles provide a milder approach for incorporating therapeutic agents into EVs while preserving their structural integrity. Although extrusion is effective for encapsulating certain cargos, it can alter the surface charge of EVs, which may influence their cellular uptake and biodistribution [98]. Similarly, freeze–thaw cycles, which are commonly employed for hydrophilic drug loading, often exhibit lower efficiency, particularly when dealing with larger or more complex macromolecules.

A promising alternative to traditional methods is cellular nanoporation, which enables the production of EVs with high payload capacities, including therapeutic RNAs and peptides. By applying electroporation to the producing cells, this technique can increase EV yield by up to 50-fold and enhance RNA loading by more than 1000-fold. This method is particularly advantageous for generating EVs from cells with low basal secretion rates, offering a scalable and efficient approach for the large-scale production of therapeutic EVs [99].

### The application of EVs in cancer immunotherapy

Due to their high accessibility, minimal immunogenicity and toxicity, intrinsic biocompatibility, and potential for targeted delivery of monotherapies or combination therapies, both natural and engineered EVs have emerged as promising candidates for cancer immunotherapy. To date, EVs have primarily been explored as DD systems, particularly in the context of immunomodulation [100]. While EVs demonstrate superior biosafety profiles compared to traditional cancer treatments, such as surgery, chemotherapy, radiation, and protein agonists, they still require extensive validation. The development of EV-based therapeutics for cancer is in its early stages, with further clinical trials expected in the future.

Currently, a significant proportion of cancer immunotherapies rely on cellular immunity mediated by T cells. This process is initiated by the release of tumor antigens, which are subsequently presented by antigen-presenting cells (APCs). The presentation of these antigens activates, recruits, and facilitates the infiltration of T cells into the TME, where activated T cells identify and destroy tumor cells. EVs have gained substantial attention for their potential to enhance the efficacy of existing cancer immunotherapies [101]. Modulating immune responses by altering signaling pathways in both cancer and immune cells has proven to be an effective strategy for cancer treatment [93]. EVs derived from T cells expressing PD-1 have demonstrated the ability to target cancer cells by binding to PD-L1 on their surface. Additionally, EVs derived from *Escherichia coli* that overexpress PD-L1 have been shown to enhance immune cell infiltration within the TME and activate CD8^+^ T cells, resulting in significant antitumor effects and a positive response to anti-PD-1 immunotherapy [102]. Furthermore, delivering EVs that overexpress PD-1 can inhibit PD-L1 expression on cancer cells, leading to the accumulation of effector T cells. This action reduces immune resistance in the TME by blocking the PD-1/PD-L1 interaction between T cells and cancer cells, thereby promoting apoptosis of cancer cells, particularly when used in combination with chemotherapy [103].

Immunosuppressive M2 macrophages are known to promote tumor growth, making their repolarization to the M1 phenotype a promising strategy for altering the TME and inhibiting tumor progression. Recent studies have shown that M1 macrophage-derived exosomes (M1-Exos) can deliver anti-PD-L1 siRNA to tumors, resulting in reduced PD-L1 expression on cancer cells and an increased population of CD8^+^ T cells. The enhanced secretion of IFN-γ from these CD8^+^ T cells, in combination with M1-Exos, further promotes the shift of macrophages from the M2 to the M1 phenotype, effectively inhibiting tumor growth [104]. Additionally, mesenchymal stem cell (MSC)-derived EVs loaded with Galectin-9 (Gal-9) siRNA can disrupt the Gal-9/dectin-1 signaling pathway. This disruption reverses immunosuppression by repolarizing M2 macrophages to M1 macrophages and recruiting cytotoxic T cells, thereby exerting an antitumor effect in pancreatic ductal adenocarcinoma (PDAC) [105]. Furthermore, EVs from M1 macrophages engineered to carry OX40 ligand (OX40L) can activate the OX40/OX40L pathway, reprogramming M2 macrophages to M1 macrophages and enhancing macrophage-mediated innate immunity [106]. Macrophage-derived EVs have also been shown to transfer GARS1, which suppresses tumor growth by inducing M1 polarization, promoting macrophage phagocytosis via activation of the RAF-MEK-ERK pathway, and triggering tumor cell death through interaction with CDH6 [107].

EVs, similar to synthetic nanovesicles, can be engineered to deliver therapeutic molecules. Tumor cells frequently evade macrophage-mediated phagocytosis by expressing CD47-binding peptides (CD47), which binds to SIRPα on macrophages. The delivery of CD47, such as RS17, via EVs can block this CD47-SIRPα interaction, thereby targeting tumor cells and modulating tumor-associated macrophage (TAM) phenotypes [108, 109]. The cytosolic RNA sensor RIG-I, when activated, can enhance the infiltration of immune cells—particularly CD4^+^ and CD8^+^ T cells—into the TME by promoting type I interferon (IFN-1) responses, leading to tumor cell apoptosis. Red blood cell (RBC)-derived EVs delivering a RIG-I agonist (immunomodulatory RNA) have been shown to diminish immunosuppressive activity and inhibit tumor growth [110]. EVs derived from CD4^+^ T cells can activate CD8^+^ T cells by transferring miRNAs, such as miR-25-3p, miR-155-5p, miR-215-5p, and miR-375, which are essential for inducing CD8^+^ T cell-mediated antitumor responses, without affecting regulatory T cells (Tregs) [111]. Additionally, EVs from activated CD8^+^ T cells can directly induce cytotoxicity against tumor cells and lead to the apoptotic elimination of mesenchymal tumor stromal cells, thereby hindering tumor progression and metastasis [112]. Chimeric antigen receptor (CAR)-T cell-derived EVs retain the specific binding and cytotoxic functions of their parent cells while reducing the risk of cytokine release syndrome (CRS) [113]. EVs equipped with anti-CD3 and anti-HER2 antibodies can recruit and activate cytotoxic T cells to target HER2-positive breast cancer (HER2^+^ BC) cells [114]. Moreover, EVs loaded with CD62L and OX40L can home to tumor-draining lymph nodes, triggering effector T cell activation and enhancing the antitumor immune response [115]. Natural killer (NK) cell-derived EVs deliver cytotoxic cargos to recipient cells, inhibiting serine/threonine protein kinase (Ser/Thr kinase) phosphorylation and inducing apoptosis in cancer cells, demonstrating effective targeting and potent therapeutic effects in both subcutaneous and orthotopic HCC mouse models [116].

### Application of engineered EVs in cancer immunotherapy

#### Engineered EVs as nanovaccine platforms for oncological immunotherapy

In recent years, tumor immunotherapy has emerged as a critical strategy in clinical cancer treatment, particularly through the use of tumor vaccines. These vaccines aim to deliver TAAs and immune-activating adjuvants to lymph nodes, where APCs process these materials to initiate T cell-mediated antitumor immunity [117]. However, conventional tumor vaccines face significant challenges, including low antigen encapsulation efficiency, poor targeting to lymph nodes, and inadequate lysosomal escape, which often result in suboptimal outcomes in both preclinical and clinical studies [118, 119]. To address these limitations, the development of nanomaterial-based carriers for tumor vaccine delivery has gained substantial attention. Nanovaccines are designed to efficiently transport antigens to lymph nodes, where they can stimulate robust antigen-specific immune responses and establish long-term immune memory. This approach helps prevent immune tolerance, reducing the risk of cancer recurrence [120]. Among the most promising tools in this area are engineered EVs, which offer unique advantages as nanovaccine delivery systems.

EVs, especially those derived from dendritic cells (DCs), have shown considerable potential in cancer immunotherapy. These EVs possess intrinsic adjuvant functions and contain natural immunogenic cargo that can activate immune responses. Furthermore, EVs can circulate throughout the body, ensuring broad distribution, making them an ideal candidate for targeted cancer immunotherapy [121, 122]. DC-derived EVs, for instance, can carry various biomolecules that are critical for stimulating T cells, and they can be engineered to deliver both nucleic acids and TAAs [123]. As such, these EVs function both as delivery vehicles and immune adjuvants, enhancing antigen presentation and promoting stronger immune responses. In contrast, tumor-derived exosomes (TEXs) are another promising source of EVs for cancer immunotherapy. TEX naturally carry substantial amounts of TAAs, making them well-suited for vaccine formulations without the need for additional antigen loading. These vesicles can transfer antigens and immunostimulatory molecules directly to DCs, activating them to mount potent immune responses. However, while TEX have great potential, they also exhibit immunosuppressive properties that can inhibit immune cell function and allow tumors to evade immune surveillance. Therefore, further studies are necessary to fully understand the effects of TEX on the immune system and how these effects can be modulated to improve therapeutic outcomes [124].

To overcome some of the challenges associated with EV-based vaccines, researchers have engineered various EVs to enhance their immunogenicity and targeting capabilities. For example, Liu et al. developed a tumor nanovaccine, HEX^@^BP, which combined black phosphorus quantum dots (BPQDs) with cancer-specific antigens and photothermal therapy (PTT). The exosomal coating not only protected BPQDs from rapid degradation but also prolonged their circulation and enhanced antigen presentation to DCs, thereby improving T cell activation and tumor targeting [125]. Similarly, Morishita et al. engineered tumor cell-derived exosomes with the pH-sensitive fusogenic peptide GALA, which facilitated the escape of tumor antigens from lysosomes and enhanced their presentation via MHC class I molecules on DCs [126]. In another notable approach, Huang et al. introduced Hela-EXOS, a vaccine incorporating human neutrophil elastase as an immunogenic cell death (ICD) inducer and hiltonol as a TLR3 agonist, which enhanced DC activity and promoted CD8^+^ T cell responses in breast cancer models [127]. Furthermore, Liu et al. demonstrated that γ-ray-irradiated glioma-derived exosomes (G-EXOs) exhibited enhanced antigenicity, effectively inducing anticancer T-lymphocyte immunity when used in DC immunotherapy vaccines [128]. Beyond antigen delivery, EV-based vaccines are being explored for their potential to modulate the TME. For instance, Lv et al. developed an exosomal vaccine derived from M1-polarized macrophages (M1OVA-Exos), which inhibited the Wnt signaling pathway and reprogrammed TAMs into the M1 phenotype. This reprogramming improved immune responses, suppressed tumor growth, and prevented metastasis [129]. These findings highlight how engineered EVs not only enhance antigen delivery but also remodel the TME to support immune activation, further boosting the efficacy of cancer immunotherapy.

**Fig. 3:**
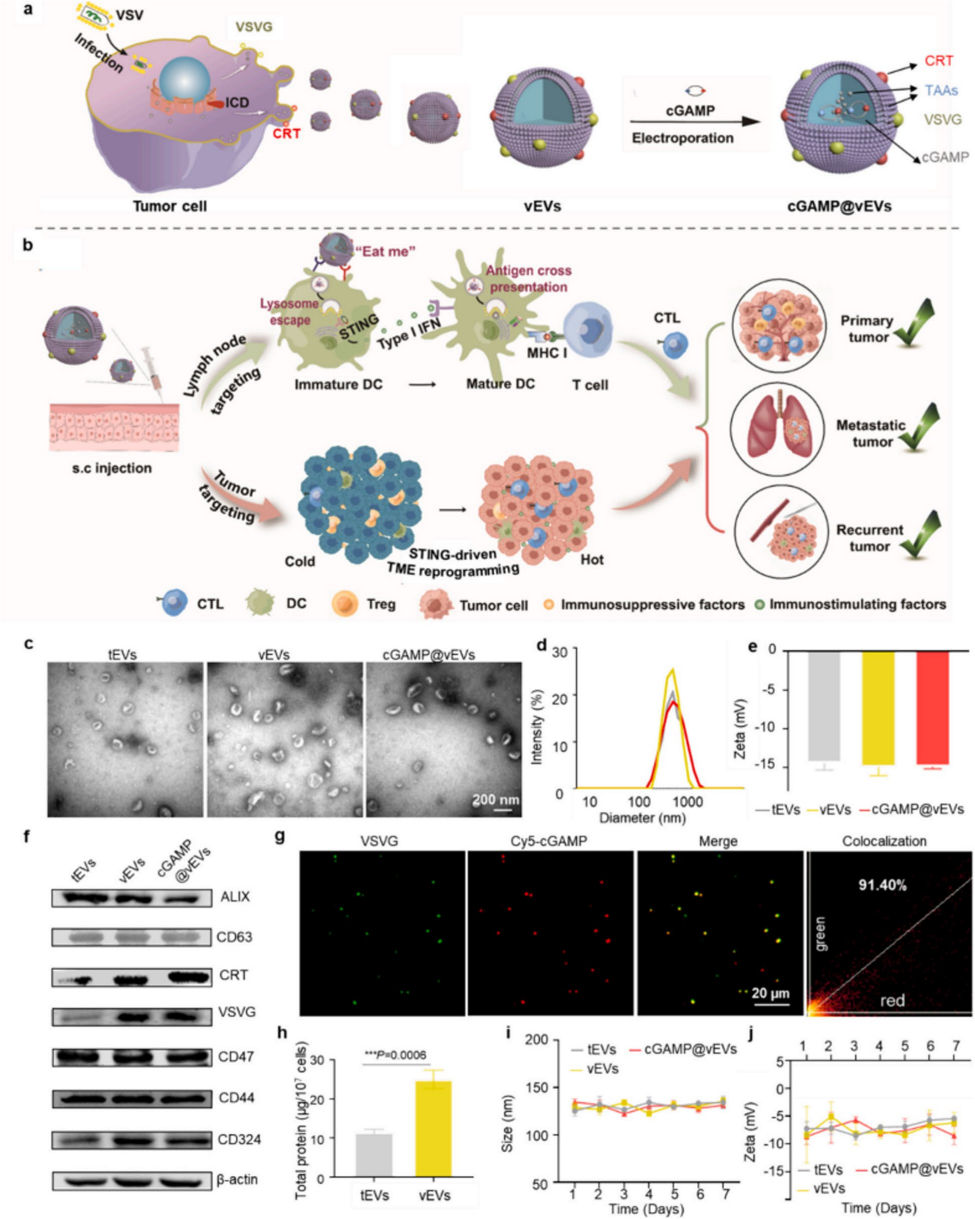
Preparation and characterization of cGAMP^@^vEVs. **a** Construction of cGAMP^@^vEVs. **b** Synergistic cancer immunotherapy mechanism of cGAMP^@^vEVs. **c** TEM imaging of the tEVs, vEVs, and cGAMP^@^vEVs. d-e, Average particle size (**d**) and ζ potential (**e**) distribution of cGAMP^@^vEVs, as analyzed by DLS. **f** Western blot analysis of CRT and VSVG expression in different EV formations. **g** Fluorescence colocalization imaging of cGAMP^@^vEVs. VSVG and cGAMP are depicted in green and red, respectively. **h** BCA protein quantification of tEVs and vEVs. **i**, **j** Size (**i**) and ζ potential (**j**) distribution of cGAMP.^@^vEVs stored in 10% FBS (v/v) for 7 days. Data are presented as mean ± S.D. (*n* = 3 biologically independent samples). Adapted with permission from Wang et al. 2025 [133]

Recent innovations have also led to the development of hybrid nanovaccines (Hy-M-Exos) combining EVs with other therapeutic modalities. For example, researchers have created DC-derived EV (DEV)-mimicking AIE nanoparticles, which integrate immune-modulating proteins and photosensitizers for photodynamic immunotherapy. This combination significantly enhances immune responses, eradicates primary tumors, and inhibits distant metastases, showing promise as an anticancer nanovaccine strategy [128]. Similarly, the use of Golgi apparatus-PD-L1-/- exosome hybrid membrane-coated nanoparticles (GENPs) has demonstrated the ability to reduce PD-L1 secretion, disrupt exosome release, and enhance systemic immune responses by activating T cells. In combination with anti-PD-L1 treatment in an in situ hydrogel, GENPs significantly lowered recurrence rates and extended survival in mice with metastatic melanoma [130]. Moreover, the creation of virus-mimicking nanovaccines has been explored to improve tumor-specific immune responses. One such example is the cGAMP^@^vEVs, a virus-mimicking nanovaccine engineered from tumor-derived vesicles (Fig. 3). This nanovaccine enhances antitumor immunity by activating the STING pathway, promoting CTL infiltration into tumors, and inhibiting tumor growth and metastasis [129]. Furthermore, the development of a DC-tumor hybrid cell-derived chimeric exosome loaded with STING agonists (DT-Exo-STING) offers a promising strategy to enhance T-cell immunity. This approach improves antigen presentation, crosses the blood–brain barrier (BBB), and transforms the immunosuppressive GBM microenvironment into a pro-inflammatory, tumoricidal state, thereby enhancing the efficacy of immune checkpoint blockade (ICB) therapy and establishing systemic immune memory [131]. Lastly, cancer vaccines targeting specific tumor types, such as head and neck squamous cell carcinoma (HNSCC), have also been enhanced by EV-based technologies. A Hy-M-Exo combining TEX with DC membrane vesicles demonstrated enhanced lymph node targeting and a robust T-cell response, providing strong therapeutic potential in a mouse model of HNSCC [132].

These findings collectively demonstrate the potential of engineered EVs as nanovaccines for cancer immunotherapy (Table 1). By overcoming the limitations of traditional tumor vaccines and offering more targeted and effective delivery, engineered EV-based vaccines are poised to play a significant role in advancing cancer treatment strategies and improving patient outcomes.

**Table 1:** Engineered EVs as nanovaccine platforms for oncological immunotherapy

Evs Type	Cancer Type	Biological role	Mechanism	Reference
DC-Derived EVs	General Cancer	Antigen delivery, immune adjuvant	Deliver antigens to dendritic cells (DCs), enhance antigen presentation, stimulate T cell activation, and promote immune responses	[123]
Tumor-Derived Exosomes (TEX)	General Cancer	Tumor antigen delivery	Carry tumor-associated antigens (TAAs), transfer them to DCs, activate immune responses. However, may also possess immunosuppressive properties that promote tumor evasion	[124]
Engineered Tumor-Derived EVs	General Cancer	Enhanced antigen presentation	Modification with fusogenic peptides (e.g., GALA) for improved lysosomal escape, enhancing antigen presentation via MHC class I on DCs	[126]
Tumor-Derived Exosomes	Breast Cancer	Immune activation and immune response promotion	Engineered with ICD inducers like human neutrophil elastase and hiltonol to boost dendritic cell (DC) activity and promote CD8^+^ T cell responses	[127]
Gamma-ray-Irradiated EVs	Melanoma	Enhanced antigenicity and T cell activation	γ-ray-irradiated exosomes increase antigenicity, enhancing anticancer T-lymphocyte immunity when incorporated into DC immunotherapy vaccines	[128]
M1-Macrophage-Derived EVs	General Cancer	Tumor microenvironment remodeling	M1-polarized macrophage-derived exosomes (M1OVA-Exos) inhibit Wnt signaling to reprogram tumor-associated macrophages (TAMs) into M1 phenotype, promoting immune responses	[129]
EVs Mimicking DCs	General Cancer	Immune modulation, photodynamic therapy (PDT)	EV-mimicking AIE nanoparticles combine immune-modulating proteins and photosensitizers for enhanced immune responses and eradication of tumors and metastases	[134]
PD-L1-/- Exosome Hybrid EVs	Metastatic Melanoma	Immune checkpoint modulation	GENPs disrupt PD-L1 secretion, activate T cells, and enhance systemic immune responses, in combination with anti-PD-L1 therapy for improved therapeutic outcomes	[130]
Virus-Mimicking Nanovaccine	General Cancer	Tumor immunotherapy through STING pathway activation	cGAMP^@^vEVs activate the STING pathway, promoting cytotoxic T lymphocyte (CTL) infiltration, inhibiting tumor growth and metastasis	[133]
DC-Tumor Hybrid EVs	Glioblastoma, Brain Cancer	Tumor-specific immunity and blood–brain barrier crossing	STING agonist-loaded chimeric exosomes (DT-Exo-STING) enhance T-cell immunity, transform the immunosuppressive glioblastoma microenvironment, and boost immune checkpoint blockade efficacy	[131]
Hybrid Nanovaccine (Tumor-Derived EVs + DCs)	Head and Neck Squamous Cell Carcinoma (HNSCC)	Antigen delivery and T cell activation	Hy-M-Exo (hybrid nanovaccine) combines tumor-derived exosomes with DC membrane vesicles to enhance lymph node targeting and T-cell responses, demonstrating significant therapeutic potential	[132]

#### Engineered EVs with cytotoxic effect for cancer immunotherapy

Adoptive immunotherapy has shown promising clinical outcomes but is often associated with the risk of systemic immune overreactions. EVs provide a safer, cell-free alternative for immunotherapy by mimicking the functions of their parent cells without inducing excessive immune responses or proliferation. This approach has gained significant interest due to its potential to offer a targeted and controlled therapeutic effect.

**Fig. 4:**
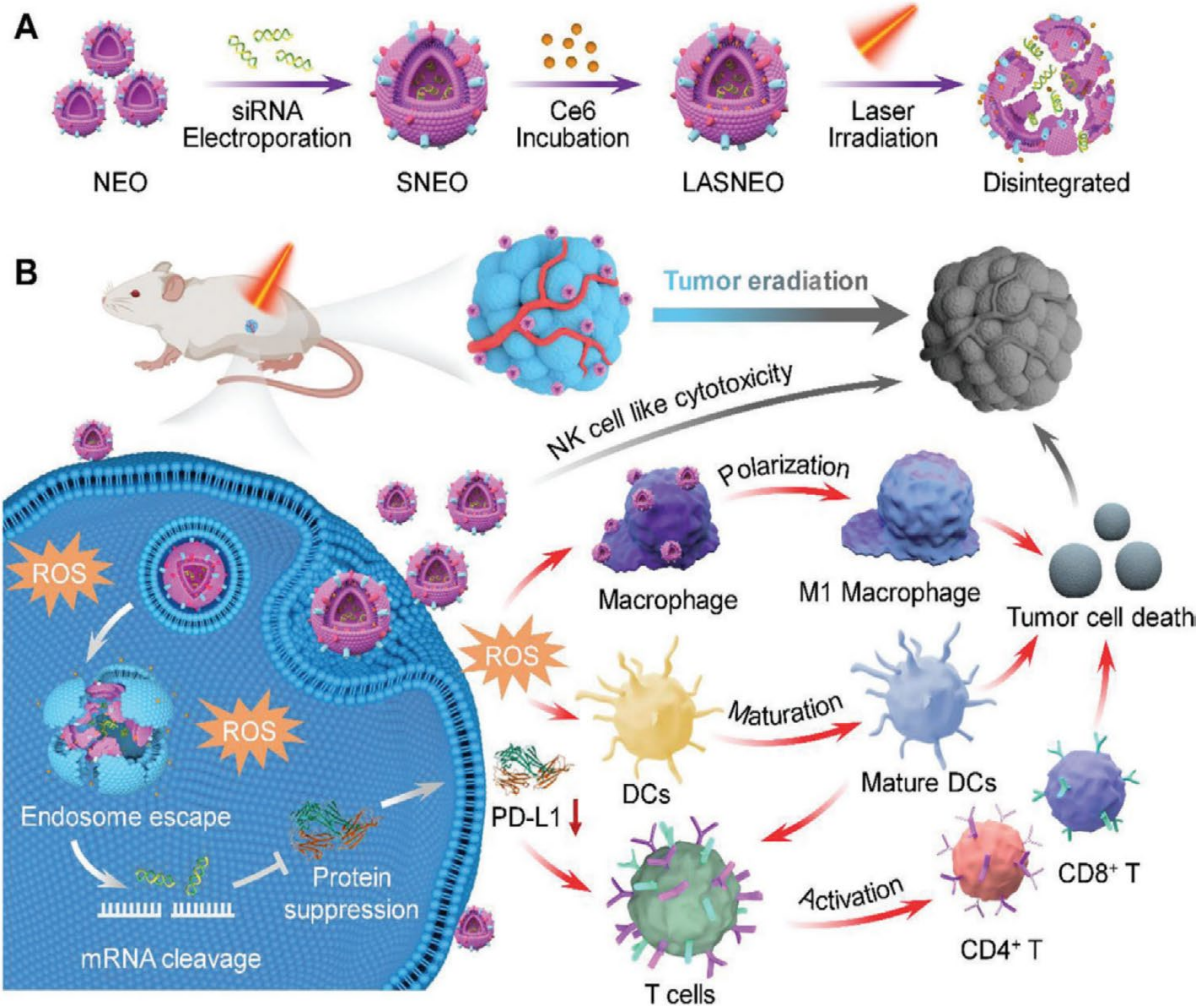
**A** Fabrication of siRNA and Ce6 dual-loaded LASNEO and the light-triggered disassemble of LASNEO. **B** Collaboratively reprogram of multiple types of immune cells by LASNEO. First, LASNEO displays NK cell like cytotoxicity. After internalized by tumor cells, LASNEOs are disintegrated under 660 nm laser irradiation and photogenerated ROS facilitates endosomal escape of siRNA, and then siRNA mediates robust gene silence of PLK1 or PD-L1. Downregulation of PD-L1 and several soluble factors contained in NEO restore T cell immune surveillance. Moreover, ROS also triggers effective photodynamic therapy and augments M1 macrophage polarization and DC maturation. Adapted with permission from Zhang et al. 2022 [139]

For instance, Wu et al. demonstrated that NK cell-derived EVs contain cytotoxic effectors such as perforin, granzyme A and B, and activated caspase proteins, providing a solid theoretical foundation for NK EV-based immunotherapy [135]. Building on this, Pitchaimani et al. engineered NK-derived EV mimetics, termed NKsome, using membrane fusion technology. These NKsome vesicles preserved NK cell membrane proteins, which enhanced their ability to naturally surveil and target mutant cells with high tumor affinity, improving tumor homing efficiency in vivo. In mouse models, NKsome exhibited substantial antitumor effects (~ 700 mm^3^ vs. ~ 900 mm^3^ in controls), and its combination with doxorubicin (Dox) further reduced tumor size to less than 200 mm^3^ [136]. Similarly, Deng et al. developed NK-derived EV mimetics by coating nanoparticles with NK cell membranes (NK-NPs). These NK-NPs demonstrated efficient tumor targeting, polarization of TAMs, and direct cytotoxicity, significantly suppressing tumor growth and metastasis in preclinical models [137]. Light-activatable silencing NK-derived exosomes (LASNEO), engineered with siRNA and photosensitizer Ce6, show potent antitumor effects by inducing NK cell-like cytotoxicity, promoting ROS-mediated photodynamic therapy, and facilitating immune cell recruitment in the tumor microenvironment, presenting significant potential for clinical applications [138] (Fig. 4).

Beyond NK cells, EVs derived from T cells and macrophages also show cytotoxic effects against tumor cells. For example, Yang et al. reported that Exos from CAR-T cells retained essential characteristics of their parent cells, including CAR expression and protein cargo. These CAR-T cell-derived Exos effectively inhibited the growth of Mesothelin-positive triple-negative breast cancer (TNBC) cells, demonstrating their potential as a novel cancer therapeutic strategy [140]. Additionally, M1 macrophage-derived biomimetic EVs have shown direct antitumor effects and can induce apoptosis in tumor cells, although the underlying mechanisms remain to be fully elucidated [141].

The potential of engineered small EVs has also been explored. For instance, one study developed small EVs with high CD47 expression and c(RGDyC) modification to deliver siRNA targeting the m6 A reader YTHDF1 for gastric cancer therapy. This platform regulated epigenetics and immune responses, suppressed tumor progression through Wnt/β-catenin pathway inactivation, and enhanced IFN-γ responses, offering a low-toxicity, highly promising immunotherapeutic approach [142]. Moreover, IL2-tethered small EVs (IL2-sEVs) derived from engineered Jurkat T cells enhanced CD8^+^ T cell anticancer activity and reduced PD-L1 expression in melanoma cells via specific microRNAs, demonstrating their potential as cancer immunotherapeutic agents [143].

Several studies also explore EVs as delivery vehicles to enhance the efficacy of existing therapies. For example, IL-12 encapsulated in EVs derived from mature DCs (DEV-IL) effectively recruited immune cells and depleted immunosuppressive cells in the tumor microenvironment, showing significant antitumor effects without the toxicity associated with free IL-12 [144]. In another study, engineered EVs modified with GE11 peptides selectively targeted EGFR-positive tumor cells and delivered Dox, showing higher targeting efficiency, increased apoptosis, and reduced cytotoxicity to normal cells compared to non-modified EVs and free Dox treatments [145]. EV-based therapies also hold promise for treating specific cancers, such as lung cancer and GBM. For lung cancer, a novel study demonstrated that EVs derived from human primary CD8^+^ T cells, containing interleukin-2 and cetuximab, exhibited enhanced antitumor activity and targeting specificity against lung cancer cells, presenting a promising therapeutic strategy [146]. In GBM, a novel EV-based nanotherapeutic enhanced by radiation therapy effectively targeted tumors, trapped TGF-β, and improved immune responses, leading to tumor regression and prolonged survival in a murine model, suggesting its potential for clinical translation [147]. Additionally, novel hybrid nanovesicles combining liposomes with exosomes from bispecific CAR-T cells (Lip-CExo^@^PTX) have been developed for lung cancer treatment. These nanovesicles improved the targeted delivery of paclitaxel (PTX), enhancing antitumor efficacy while reducing systemic toxicity and prolonging survival in a metastatic lung cancer model [148].

Collectively, these studies highlight the versatility and therapeutic potential of EVs, particularly those engineered from immune cells, in cancer immunotherapy (Table 2). While the cytotoxic mechanisms of immune cell-derived EVs significantly broaden the scope of cell-free immunotherapy, further research is required to fully elucidate the precise mechanisms behind these effects and optimize their clinical application.

**Table 2:** Engineered EVs with cytotoxic effect for cancer immunotherapy

Evs Type	Cancer Type	Biological role	Mechanism	Reference
NK cell-derived EVs	General (tumors)	Cytotoxicity against tumor cells	Contains perforin, granzyme A/B, and caspase proteins, mediating direct cytotoxicity	[135]
NK-derived EV mimetics (NKsome)	General (tumors)	Tumor targeting and antitumor activity	NK cell membrane proteins enhance tumor homing efficiency, synergize with doxorubicin	[136]
NK-derived EV mimetics (NK-NPs)	General (tumors)	Tumor targeting, immune modulation, cytotoxicity	Tumor-associated macrophage polarization, direct cytotoxicity	[137]
CAR-T cell-derived Exosomes	Mesothelin-positive triple-negative breast cancer (TNBC)	Inhibition of tumor cell growth	Retain CAR expression and protein cargo to target TNBC cells	[140]
M1 macrophage-derived EVs	General (tumors)	Antitumor effects, apoptosis induction	Direct cytotoxicity, inducing apoptosis in tumor cells	[141]
Small engineered EVs	Gastric cancer	Delivery of siRNA targeting m6 A reader YTHDF1	Modulates Wnt/β-catenin pathway, enhances IFN-γ response, low toxicity	[142]
IL2-tethered small EVs	Melanoma	Enhancement of CD8 + T cell anticancer activity	Reduces PD-L1 expression, induces specific microRNA activity	[143]
Dendritic cell-derived EVs (DEV-IL)	General (tumors)	IL-12 delivery to recruit immune cells, deplete immunosuppressive cells	Recruits immune cells, reduces tumor immune suppression, enhances antitumor activity	[144]
Engineered EVs with GE11 peptides	EGFR-positive tumors (General)	Targeting EGFR-positive tumor cells, drug delivery	Selective targeting of EGFR-positive cells, enhances apoptosis, reduces toxicity	[145]
CD8^+^ T cell-derived EVs	Lung cancer	Enhanced antitumor activity and specificity	Interleukin-2 and cetuximab enhance targeting and immune response	[146]
Radiation-enhanced EVs	Glioblastoma	Tumor regression and survival prolongation	Traps TGF-β, improves immune responses, tumor targeting	[147]
Hybrid nanovesicles (Lip-CExo^@^PTX)	Lung cancer	Targeted delivery of paclitaxel (PTX)	Reduces systemic toxicity, enhances antitumor efficacy and survival	[148]

#### Engineered EVs for remodeling the tumor immune microenvironment

The tumor immune microenvironment (TIME) is often characterized by immune suppression, which presents a significant barrier to the effective activation of immune responses during cancer immunotherapy [149]. One of the most promising strategies to overcome this immunosuppressive microenvironment is the reprogramming of the TIME, aiming to enhance the efficacy of cancer immunotherapy. EVs, naturally derived from cells and capable of transferring functional biomolecules such as nucleic acids and proteins, have gained considerable attention for their potential in immune modulation and reprogramming.

In this context, several studies have demonstrated the ability of EVs to influence the phenotype and function of TAMs, a key component of the TIME. Wang et al. showed that Exos derived from M1 macrophages could promote TAMs to adopt a pro-inflammatory phenotype, thereby enhancing the antitumor effects of chemotherapy [150]. This observation underscores the critical role that M1-type EVs can play in reshaping the tumor immune landscape. Expanding on this, Ding et al. engineered stimuli-responsive M1-type EVs that could induce the expression of CD86 (a marker for M1 macrophages) and suppress CD206 (an M2 macrophage marker), resulting in enhanced pro-inflammatory cytokine secretion, including IL-6, TNF-α, and H2O2 [151]. These findings further support the potential of M1-type EVs as immune modulators that can effectively reprogram the TIME.

However, the inherent heterogeneity of EVs, influenced by different isolation methods, presents a significant challenge. To address this, Choo et al. developed homogeneous biomimetic EVs and demonstrated their ability to reprogram TAMs to the M1 phenotype both in vitro and in vivo, thereby significantly inhibiting tumor growth. When combined with immune checkpoint inhibitors (ICIs), these M1-type nanovesicles (M1 NVs) achieved exceptional therapeutic outcomes, highlighting their potential as powerful modulators of the TIME [152]. Despite these advances, the specific regulatory mechanisms of M1-derived EVs in immune system remodeling remain incompletely understood, which poses a barrier to their widespread clinical application.

Beyond reprogramming TAMs, EVs are increasingly being utilized as delivery vehicles for immune modulators, offering a potent strategy to reshape the TIME. For instance, Peng et al. demonstrated that Exos could effectively deliver RIG-1 agonists for anticancer immunotherapy. This strategy activated the RIG-1 pathway, triggering the release of IFNs and promoting immune activation against cancer cells [110]. Similarly, Jang et al. employed Exos derived from HEK293 cells to deliver STING agonists, which enhanced intratumoral retention of cyclic dinucleotides (CDNs), thereby activating the cGAS-STING pathway and inducing strong antitumor immune responses while reducing systemic inflammation [153]. These studies illustrate the potential of engineered EVs as delivery systems that can enhance immune activation within the tumor microenvironment.

In addition to immune modulation, EVs have also been explored for their role in inducing ICD, a process that can convert “cold” tumors into “hot” tumors, thereby making them more susceptible to immune attacks. Zhang et al. targeted GBM, a highly immunosuppressive tumor, using endothelial cell-derived Exos to deliver Dox, which successfully induced ICD and crossed the BBB, ultimately improving survival in GBM-bearing mice [149]. This study highlights the potential of Exos as delivery vehicles to overcome physiological barriers, such as the BBB, in cancer therapy. Similarly, Zhou et al. used Exos derived from bone marrow mesenchymal stem cells (BM-MSCs) to deliver the ICD inducer oxaliplatin and Gal-9 siRNA to PDAC. Their results demonstrated that these Exos not only enhanced drug targeting but also promoted the reprogramming of the PDAC-TME by recruiting cytotoxic T cells, repolarizing macrophages, and reducing Tregs, which further supports the potential of Exos in immune reprogramming [105].

Moreover, engineered EVs are also being utilized to counteract immune evasion mechanisms in cancers. In the case of Epstein-Barr virus (EBV)-positive nasopharyngeal carcinoma (NPC), research has shown that latent membrane protein 1 (LMP1) promotes immune evasion by recruiting ALIX to load PD-L1 into small EVs, thereby inhibiting CD8^+^ T cell function and enhancing tumor immunosuppression. Targeting the LMP1-ALIX-PD-L1 axis represents a promising strategy for improving immune responses in NPC [154]. Similarly, PDAC cells evade immune detection through CAF-derived EVs carrying long non-coding RNA RP11-161H23.5, which downregulates HLA-A expression and impairs antigen presentation. By using engineered EVs to deliver small interfering RNAs (siRNAs) targeting this pathway, the immune evasion mechanism can be counteracted, thereby improving the effectiveness of immunotherapy in PDAC [155].

**Fig. 5:**
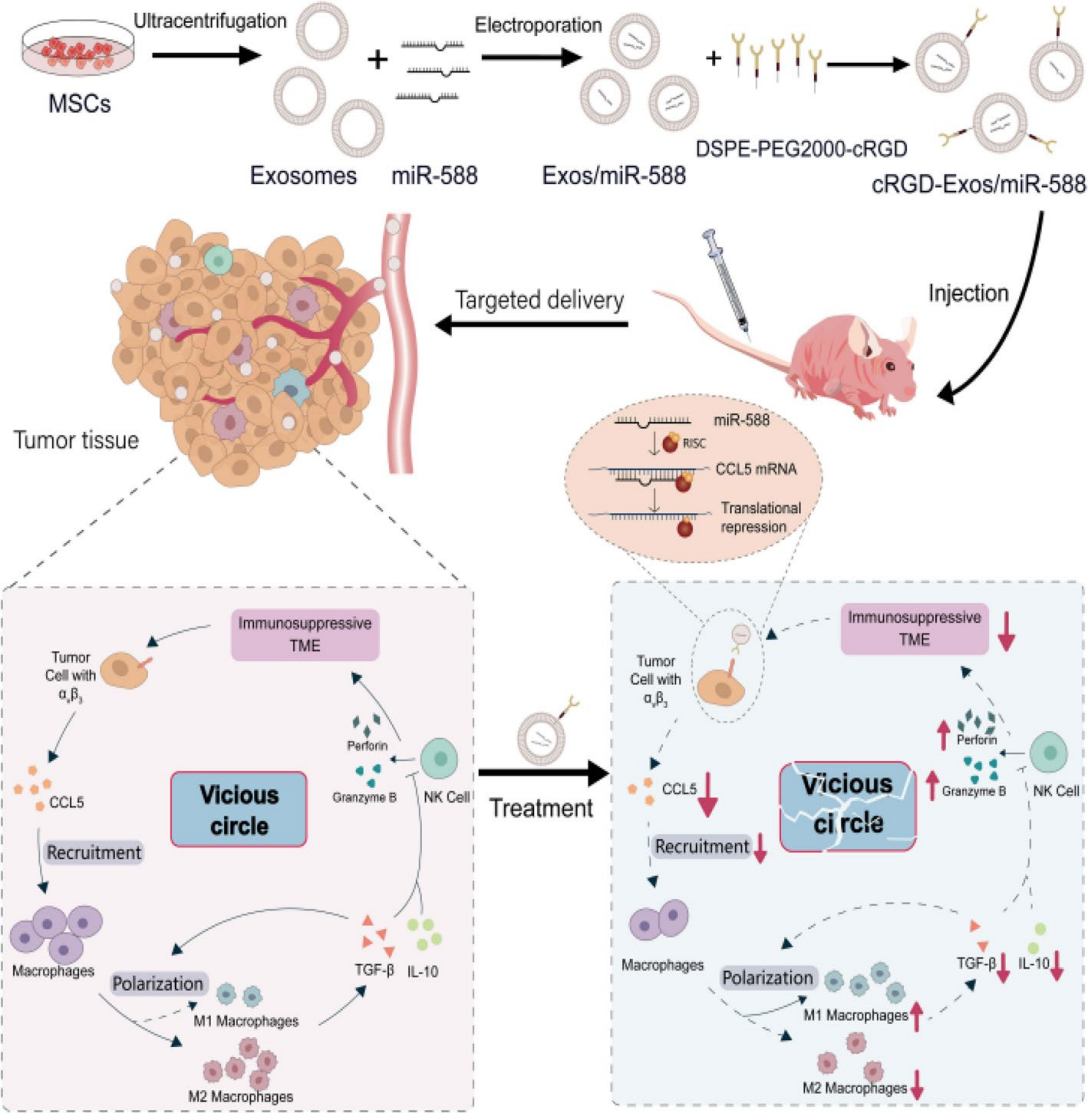
Schematic illustration of the synthesis of cRGD-Exos/miR-588 and the mechanism of anti-tumor, remodeling effect on the immunosuppressive TME. Adapted with permission from Zhang et al. 2024 [157]

In addition to immune modulation, genetic engineering of EVs has been utilized to enhance their therapeutic potential. For instance, an exosomal antibody surface display platform (LEAP) has been developed, enabling the presentation of single chain variable fragments (scFvs) on EVs. Applied to HEK293 T cell-derived EVs, this platform showed promise in eliciting T-cell anti-tumor immunity, indicating the broad applicability of EVs in cancer immunotherapy [82]. Similarly, a novel study demonstrated the potential of multifunctional hybrid exosomes targeting the cGAS-STING pathway. These hybrid exosomes, combining properties of tumor-derived CD47 and M1 macrophage exosomes, were loaded with SN38 and MnO2 to induce DNA damage, stimulate innate immunity, and promote immune cell infiltration, leading to significant anti-tumor and anti-metastatic effects [156]. Zhang et al. developed cRGD-modified exosomes for targeted delivery of miR-588, demonstrating significant tumor targeting and efficacy in remodeling the TIME in triple-negative breast cancer (TNBC) by anchoring CCL5. The results suggest that cRGD-Exos/miR-588 offers a promising approach for TNBC treatment through effective TME targeting and miRNA-based antitumor therapy (Fig. 5) [157].

Finally, in the context of radiotherapy, a novel approach involves the use of engineered RT-induced microparticles (RT-MPs) combined with immune modulators such as tIL-15/tCCL19 and PD-1 monoclonal antibodies. This combination effectively activates antitumor immune responses and significantly prolongs survival in mouse models, offering a promising alternative for cancers resistant to conventional radiotherapy [158]. Moreover, the combination of engineered EVs with ICD inducers and ICIs presents an exciting opportunity for enhancing cancer immunotherapy outcomes, especially in immunosuppressive microenvironments.

In conclusion, engineered EVs present a versatile and potent strategy for remodeling the TIME (Table 3). By reprogramming immune cells, enhancing drug delivery, and overcoming immune evasion mechanisms, EV-based therapies hold great promise in improving cancer immunotherapy. However, further research is needed to fully elucidate the underlying mechanisms of EV-mediated immune modulation and to optimize their clinical translation.

**Table 3:** Engineered EVs for remodeling the tumor immune microenvironment

Evs Type	Cancer Type	Biological role	Mechanism	Reference
Exosomes derived from M1 macrophages	Not specified	Reprogram tumor-associated macrophages (TAMs) to pro-inflammatory phenotype	M1-derived exosomes promote TAMs to adopt a pro-inflammatory phenotype, enhancing the antitumor effects of chemotherapy	[150]
Stimuli-responsive M1-type EVs	Not specified	Enhance immune response by promoting M1 macrophage polarization	M1-type EVs induce the expression of CD86 and suppress CD206, leading to enhanced pro-inflammatory cytokine secretion (IL-6, TNF-α, H2O2)	[151]
Homogeneous biomimetic M1-type nanovesicles (M1 NVs)	Not specified	Reprogram TAMs to M1 phenotype and inhibit tumor growth	Biomimetic M1 NVs reprogram TAMs to M1 phenotype in vitro and in vivo, synergizing with immune checkpoint inhibitors (ICIs) to achieve enhanced therapeutic outcomes	[152]
Exosomes delivering RIG-1 agonists	Not specified	Activate immune response via RIG-1 pathway	RIG-1 agonists in exosomes activate the RIG-1 pathway, inducing the release of interferons (IFNs) to promote immune activation against cancer cells	[110]
Exosomes delivering STING agonists	Not specified	Enhance antitumor immune response via cGAS-STING pathway	Exosomes from HEK293 cells deliver STING agonists, enhancing intratumoral retention of cyclic dinucleotides (CDNs), activating the cGAS-STING pathway and inducing antitumor immunity	[153]
Exosomes from endothelial cells delivering doxorubicin	Glioblastoma	Induce immunogenic cell death (ICD) and cross blood–brain barrier	Endothelial-derived exosomes deliver doxorubicin, inducing ICD and crossing the blood–brain barrier, improving survival in glioblastoma-bearing mice	[149]
Exosomes from BM-MSCs delivering oxaliplatin and Gal-9 siRNA	Pancreatic ductal adenocarcinoma (PDAC)	Enhance immune reprogramming and recruit cytotoxic T cells	BM-MSC-derived exosomes deliver oxaliplatin and Gal-9 siRNA, enhancing drug targeting and promoting reprogramming of the PDAC-TME by recruiting cytotoxic T cells and repolarizing TAMs	[105]
EVs targeting LMP1-ALIX-PD-L1 axis	Nasopharyngeal carcinoma (NPC)	Overcome immune evasion and enhance immune responses	EVs target the LMP1-ALIX-PD-L1 axis to counteract immune evasion in EBV-positive NPC by inhibiting PD-L1-mediated immune suppression and restoring CD8^+^T cell function	[154]
EVs delivering siRNAs targeting RP11-161H23.5	Pancreatic ductal adenocarcinoma (PDAC)	Overcome immune evasion by downregulating HLA-A	CAF-derived EVs deliver siRNAs targeting RP11-161H23.5, reversing HLA-A downregulation and improving antigen presentation to enhance immunotherapy effectiveness	[155]
Engineered exosomal antibody surface display platform (LEAP)	Not specified	Elicit T-cell anti-tumor immunity	LEAP platform presents scFvs on EVs, eliciting T-cell-mediated anti-tumor immunity, enhancing cancer immunotherapy efficacy	[82]
Multifunctional hybrid exosomes targeting cGAS-STING pathway	Not specified	Induce DNA damage, stimulate innate immunity, and promote immune cell infiltration	Hybrid exosomes combining tumor-derived CD47 and M1 macrophage exosomes deliver SN38 and MnO2 to induce DNA damage, activate innate immunity, and stimulate immune cell infiltration	[156]
Engineered RT-induced microparticles (RT-MPs) combined with immune modulators	Not specified	Enhance radiotherapy response and activate antitumor immunity	RT-MPs combined with tIL-15/tCCL19 and PD-1 monoclonal antibodies activate antitumor immune responses, significantly prolonging survival in mouse models resistant to radiotherapy	[158]

#### Engineered EVs as immune checkpoint blocking agent for cancer immunotherapy

ICB therapy, particularly targeting PD-1 and PD-L1, has become a cornerstone in cancer treatment, revolutionizing immunotherapy. Despite its success, the standalone efficacy of ICB is often limited due to the immunosuppressive TME. To enhance the effectiveness of ICB therapy, recent studies have explored the potential of EVs as engineered delivery vehicles for ICB. These studies utilize various EV types, including T-cell-derived vesicles, bacterial outer membrane vesicles (OMVs), and TEX, to improve tumor targeting, modulate immune responses, and block checkpoint pathways.

One of the first significant advancements in this field came from Han et al. [159], who engineered PD1-enriched EV mimetics by using T cell membranes. These EV mimetics were designed to enhance tumor targeting through immune recognition, and they demonstrated an increase in nanoparticle accumulation in tumors, thereby improving the efficacy of PTT without adverse effects. However, the study did not specifically evaluate the immunotherapeutic impact of these PD1-enriched EV mimetics, leaving room for further investigation into their potential for checkpoint blockade. Furthering this approach, Chen et al. [93] genetically engineered PD1-enriched EVs as a strategy for cancer therapy. By knocking out PD-L1 and HLA-I, they aimed to reduce the immune suppression commonly induced by TEXs. Their results revealed that PD1-enriched EVs were as effective as clinical monoclonal antibodies targeting PD1, suggesting that engineered EVs expressing PD1 could simultaneously promote immune recognition and block checkpoint inhibition, offering a promising alternative to traditional antibody-based therapies.

Bacterial OMVs, naturally endowed with immune-activating properties, have also been engineered to express PD1 for checkpoint blockade. Li et al. [103] modified OMVs to display the external domain of PD1 on their surfaces, adding a functional ICB mechanism without compromising their inherent immune-stimulating abilities. In animal models, PD1-OMVs showed superior anticancer effects compared to PD-L1 blockade alone or OMVs combined with anti-PD-L1 antibodies, highlighting the potential of OMVs as multifunctional platforms for enhancing ICB therapy. In a similar vein, Pan et al. [160] developed OMVs engineered to deliver PD1 plasmids directly to tumor cells, aiming to induce autologous PD-L1 blockade within the TME. By modifying OMVs with LyP1 polypeptides, they enhanced tumor targeting and facilitated the delivery of PD1-encoding plasmids to tumor cells. These engineered OMVs not only recruited cytotoxic T lymphocytes (CTLs) to the tumor site but also induced interferon-gamma (IFN-γ) secretion, resulting in improved anticancer effects in a mouse colon cancer model. This approach demonstrated that OMVs could effectively enhance ICB and promote stronger antitumor immunity.

**Fig. 6:**
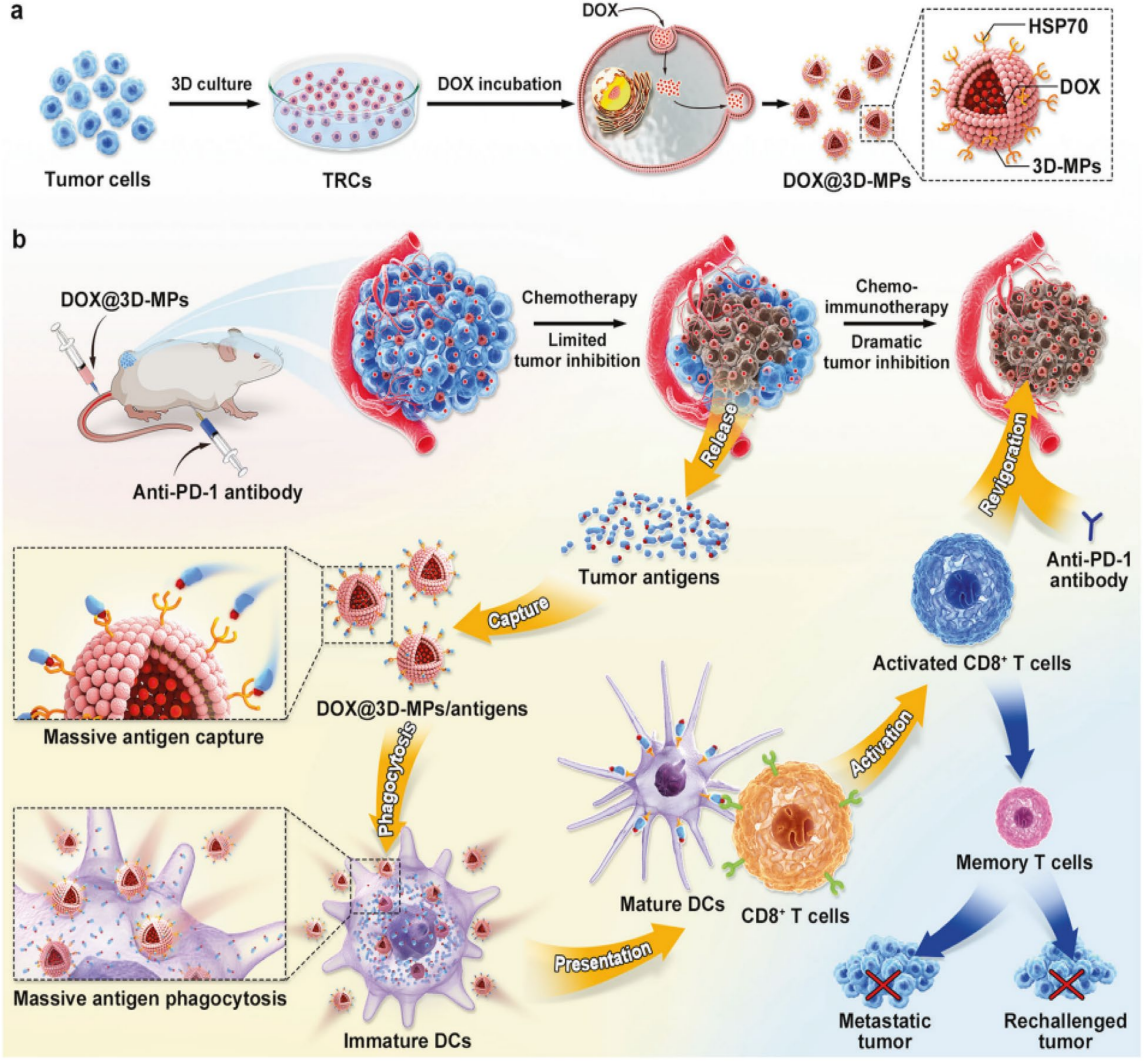
a Schematic illustration of the preparation of DOX^@^3D-MPs. b Schematic illustration of DOX^@^3D-MPs to boost anti-PD-1 antibody therapy. DOX^@^3D-MPs efficiently targeted tumor cells and induced ICD of tumor cells to release enough tumor antigens. 3D-MPs assisted capturing tumor antigens to promote their phagocytosis by DCs and the subsequent DC maturation and CD8^+^ T cell activation in a HSP70-dependent manner. Combination of DOX.^@^3D-MPs and antiPD-1 antibody efficiently hindered tumor growth, generating strong antitumor immune memory to restrain tumor relapse and metastasis. Adapted with permission from Bie et al. 2023 [161]

The application of EVs as immune checkpoint modulators extends beyond PD1 and PD-L1 inhibition. For example, tumor-repopulating cell-derived microparticles loaded with Dox (DOX^@^3D-MPs) have been developed to enhance ICB by inducing ICD, promoting antigen presentation by DCs, and activating CD8^+^ T cells. These particles demonstrated significant improvements in therapeutic responses across various tumor models and induced long-lasting immune memory [161]. This study emphasizes the potential of engineered EVs to not only block immune checkpoints but also to actively enhance immune system activation (Fig. 6).

On the other hand, TEXs, which are known to hinder the effectiveness of ICB therapies, have been targeted to improve immunotherapy outcomes. One approach involves utilizing an engineered antiviral peptide to disrupt T-EXOs, thereby enhancing CD8^+^ T cell function and reshaping the TME. This strategy has shown promise in preventing premetastatic niche formation and improving therapeutic efficacy in treated mice [162]. These findings highlight the importance of targeting tumor-derived vesicles to overcome immune suppression and improve the outcomes of checkpoint blockade therapies. In addition to enhancing immune responses, engineered EVs have been developed to address some of the adverse effects of ICB therapy. A study by Zhao et al. [163] proposed a BioMeder strategy utilizing bioorthogonal metabolic engineering to redirect tumor-derived extracellular vesicles (TuEVs) with myocardial-targeting peptides. This approach mitigated ICI-induced cardiotoxicity by increasing PD-L1 levels in cardiomyocytes, inhibiting T-cell activity, and thereby enhancing the safety profile of ICB therapies. This strategy has also shown promise in preventing ICI-induced type 1 diabetes, offering a versatile solution for managing ICI-related adverse events. Another novel approach involves the use of engineered EVs as ultrasound contrast agents for both imaging and checkpoint blockade. Gp-EVtPD1, an EV-based contrast agent, was developed to display truncated PD1 on its surface and encapsulate it in calcium bicarbonate (Ca(HCO3)2) particles. This agent not only enables non-invasive imaging of PD-L1 in tumor tissues but also boosts anti-tumor immunity by inducing PD-L1 degradation within endosomes and lysosomes. This dual functionality of Gp-EVtPD1 further demonstrates the potential of EVs in both diagnostic and therapeutic applications [164].

As ICB therapies continue to show efficacy in various cancers, particularly in non-small-cell lung cancer (NSCLC), there is increasing interest in utilizing EVs as a tool for monitoring treatment response. A study by Zhang et al. [165] developed a mesoporous gold sensor (MGS) assay to evaluate the phosphorylation status of PD-L1 in plasma EVs (EV pPD-L1), which correlates with tumor PD-L1 expression in tissue biopsies. This assay offers a rapid and reliable method for patient selection and monitoring of ICB therapy, further emphasizing the diagnostic potential of EVs in personalized cancer treatments.

Recent advances in therapeutic EVs, such as bispecific EVs (BsEVs), also show great promise in improving cancer immunotherapy. BsEVs are engineered to target tumor antigens and block immune checkpoint proteins simultaneously. These vesicles have demonstrated significant potential in reversing immunosuppressive landscapes within the TME, preventing premetastatic niche formation, and enhancing tumor targeting, making them a strong candidate for personalized cancer treatments [166]. Moreover, research has explored the use of probiotics in conjunction with EVs to improve ICB outcomes. Extracellular vesicles derived from *Lactobacillus rhamnosus GG* have been shown to modulate intestinal immunity, increase beneficial gut microbiota, and enhance anti-PD-1 therapy efficacy in colorectal cancer models, suggesting a novel avenue for improving cancer immunotherapy through microbiome modulation [167]. Finally, the role of PD-L1 in TAMs has been extensively studied in the context of immunosuppression. Our own research highlights the potential of targeted milk exosomes to deliver siPDL1 specifically to M2 TAMs, repolarizing them to the M1 phenotype and restoring CD8^+^ T cell activity. This targeted delivery system resulted in significant tumor growth inhibition in a mouse model of EGFR^+^ cancer, suggesting that PD-L1 targeting in TAMs offers a promising therapeutic strategy for enhancing ICB efficacy [168].

The engineering of EVs to block immune checkpoints represents an innovative and multifaceted approach to enhancing cancer immunotherapy (Table 4). By modulating immune responses, enhancing tumor targeting, and overcoming immunosuppressive barriers in the TME, engineered EVs show great promise in improving the efficacy and safety of ICB therapies. Continued research into optimizing the design and delivery of these vesicles will likely pave the way for more effective and personalized cancer treatments.

**Table 4:** Engineered EVs as immune checkpoint blocking agent for cancer immunotherapy

Evs Type	Cancer Type	Biological role	Mechanism	Reference
T-cell-derived EV mimetics	Not specified	Enhance tumor targeting, improve photothermal therapy efficacy	PD1-enriched T-cell membrane EV mimetics increase tumor accumulation and immune recognition	[159]
Genetically engineered PD1-enriched EVs	Not specified	Immune checkpoint blockade, promote immune recognition	Engineered PD1-enriched EVs to reduce immune suppression by tumor-derived exosomes (TEXs)	[93]
Bacterial outer membrane vesicles (OMVs)	Not specified	Enhance immune activation, PD1 blockade	Engineered OMVs expressing PD1 external domain, maintaining immune-stimulating properties	[103]
Bacterial outer membrane vesicles (OMVs)	Mouse colon cancer	Enhance tumor targeting and immune response	LyP1-modified OMVs deliver PD1-encoding plasmids to tumor cells, recruit CTLs, and induce IFN-γ	[160]
Tumor-repopulating cell-derived microparticles (DOX^@^3D-MPs)	Various tumor types	Enhance ICB therapy efficacy, induce immunogenic cell death	Delivering doxorubicin (DOX) to promote antigen presentation and CD8^+^ T cell activation, enhancing immune memory	[161]
Tumor-derived exosomes (T-EXOs)	Not specified	Improve ICB therapy outcomes, enhance CD8^+^ T cell function	Disrupting T-EXOs with antiviral peptide, improving immune microenvironment, preventing premetastatic niche formation	[162]
Tumor-derived EVs (TuEVs)	Not specified	Reduce ICI-related cardiotoxicity, enhance ICI therapy safety and efficacy	Bioorthogonal metabolic engineering to couple myocardial-targeting peptides with PD-L1 on TuEVs, inhibiting T-cell activity	[163]
Ultrasound contrast agent EVs (Gp-EVtPD1)	Not specified	Enhance anti-tumor immune response, improve therapeutic efficacy	EVs displaying truncated PD1 and encapsulated in Ca(HCO3)2, used for imaging and PD-L1 degradation, promoting anti-tumor immunity	[164]
Plasma EVs (EV pPD-L1)	Non-small-cell lung cancer (NSCLC)	Rapid assessment of PD-L1 phosphorylation status, assist patient selection	Measuring phosphorylation status of PD-L1 in plasma EVs (EV pPD-L1) for clinical selection	[165]
Bispecific EVs (BsEVs)	Not specified	Target tumor antigens and immune checkpoint proteins simultaneously, enhance tumor targeting	Engineered bispecific EVs to target tumor antigens and block immune checkpoints, reversing immunosuppressive landscape	[166]
Probiotic-derived EVs	Colorectal cancer	Improve anti-PD-1 immunotherapy efficacy, modulate gut immunity	EVs from Lactobacillus rhamnosus GG improving gut immunity, enhancing anti-PD-1 therapy efficacy	[167]
Milk-derived EVs	EGFR^+^ tumors	Restore CD8^+^ T cell activity, inhibit tumor growth	Targeting M2 tumor-associated macrophages, delivering siPDL1 to repolarize to M1 phenotype, restoring immune response and inhibiting tumor growth	[168]

#### Engineered EVs with stimuli-responsive properties for precision cancer immunotherapy

Precision immunotherapy aims to minimize adverse reactions by delivering highly specific treatments to tumors. One promising strategy to achieve this is the incorporation of stimuli-responsive elements into EVs, which allow for controlled drug release in response to both internal and external triggers. The TME often exhibits unique characteristics, such as altered pH, redox states, and gene expression, which can be exploited as endogenous stimuli for targeted therapies. For example, Kim et al. engineered exosomes to express a fusion gene encoding a mutant viral glycoprotein (VSV-G) on their membranes, creating mVSVG-Exos. These engineered exosomes deliver the viral antigen to cancer cells in a pH-dependent manner, enhancing tumor immunogenicity by presenting pathogen-associated molecular patterns (PAMPs), which the immune system recognizes as foreign and triggers an immune response [169].

In addition to exploiting endogenous stimuli, exogenous triggers such as light, heat, ultrasound, and magnetic fields (MF) have been widely studied for cancer treatment. Lv et al. developed hybrid nanoparticles (HNPs) by combining genetically engineered EVs with thermosensitive liposomes (TSL). These nanoparticles effectively penetrate tumor tissues, specifically in peritoneal carcinomatosis (MPC), and release their therapeutic payload in response to hyperthermic intraperitoneal chemotherapy (HIPEC) at lower temperatures, addressing the challenge of inefficient drug penetration in such therapies [170]. Similarly, Cheng et al. engineered hybrid therapeutic nanovesicles (hGLVs) by merging gene-engineered exosomes (GE-Exosomes) with drug-loaded TSL. These hGLVs, which overexpress CD47, exhibited extended circulation times and enhanced macrophage-mediated phagocytosis of tumor cells by inhibiting CD47 signaling. Moreover, photothermal stimuli were employed to trigger drug release, further improving treatment efficacy [109]. These innovations underscore the potential of stimuli-responsive EVs in precision cancer immunotherapy.

Notably, the BBB poses a significant challenge in treating glioblastoma multiforme (GBM), a highly invasive and treatment-resistant brain cancer. To address this, recent studies have introduced biomimetic approaches, such as genetically engineered exosome nanocatalysts (Mn^@^Bi2Se3^@^RGE-Exos), which enhance BBB penetration and exhibit enzyme-like activities when exposed to near-infrared (NIR) light. This approach significantly improves oxidative stress-induced GBM cell damage, demonstrating promise in anticancer therapy with favorable biosafety profiles [171]. Furthermore, stimuli-responsive EVs are not limited to cancer therapy alone. For instance, a study aimed at diabetic wound healing developed a system where adipose-derived stem cell exosomes were encapsulated within Ag^@^BSA nanoflowers and injectable collagen hydrogel. This system targets the oxidative wound microenvironment, ensuring controlled release of therapeutic agents that enhance bacterial elimination, cell apoptosis, and tissue regeneration, ultimately promoting neovascularization and improving wound healing in diabetic models [172]. Finally, engineering EVs for targeted therapy extends to hematological malignancies. An innovative approach using B-cell-derived EVs loaded with zinc oxide nanocrystals (ZnO NCs) and anti-CD20 monoclonal antibodies has been developed for lymphoma treatment. This system, termed TrojanNanoHorse (TNH), demonstrated high biocompatibility and targeting specificity. When activated by high-energy ultrasound shock waves, TNH significantly enhanced cytotoxicity against CD20^+^ lymphoid cancer cells, highlighting the versatility and efficacy of engineered EVs in both solid and hematological cancers [173].

In addition to these innovations, dendritic cell-derived exosomes (DEXs) are also gaining attention in cancer immunotherapy [174]. DEXs carry common immune-stimulatory molecules, including MHCI, MHCII, CD80, CD86, and CD40. Compared to traditional dendritic cell-based immunotherapy, DEXs have advantages in large-scale production, storage, and quality control. Due to their expression of MHC molecules, DEXs are capable of regulating various cellular activities. Their immunotherapy mechanisms include loading tumor-associated antigens and expressing anti-tumor immunity after loading. In addition, DEXs express adhesion molecules (such as ICAM-1 and MFG-E8) and membrane-stimulating proteins (such as TNF), which help activate and secrete NK cells to fight against cancer. Clinical trials have shown promising results in utilizing DEXs for cancer immunotherapy, demonstrating their potential in enhancing immune responses and targeting tumor cells more effectively (NCT01159288).

These advancements illustrate the immense potential of engineered, stimuli-responsive EVs in advancing precision immunotherapy (Table 5). By combining endogenous and exogenous triggers with innovative EV design, these therapies promise to offer more targeted and effective treatments for various cancer types, as well as other diseases requiring precision therapy.

**Table 5:** Engineered EVs with stimuli-responsive properties for precision cancer immunotherapy

Evs Type	Cancer Type	Biological role	Mechanism	Reference
Exosomes expressing a mutant viral glycoprotein (VSV-G)	Not specified	Enhance tumor immunogenicity via pathogen-associated molecular patterns (PAMPs)	pH-dependent delivery of viral antigen to cancer cells, triggering an immune response through recognition of PAMPs	[169]
Hybrid nanoparticles (HNPs) combining EVs and thermosensitive liposomes	Peritoneal carcinomatosis (MPC)	Enhance drug penetration and release via hyperthermic intraperitoneal chemotherapy (HIPEC)	Thermosensitive liposomes release therapeutic payload when exposed to hyperthermic conditions, improving drug penetration	[170]
Hybrid therapeutic nanovesicles (hGLVs) combining GE-Exosomes and thermosensitive liposomes	Not specified	Improve macrophage-mediated phagocytosis and drug release via photothermal stimuli	Overexpression of CD47 on hGLVs inhibits CD47 signaling, improving macrophage-mediated phagocytosis; photothermal stimulus triggers drug release	[109]
Genetically engineered exosome nanocatalysts (Mn^@^Bi2Se3^@^RGE-Exos)	Glioblastoma (GBM)	Enhance BBB penetration and oxidative stress-induced cell damage	NIR-II light triggers enzyme-like activities in Mn@Bi2Se3@RGE-Exos, improving GBM cell damage and oxidative stress response	[171]
Adipose-derived stem cell exosomes encapsulated within Ag^@^BSA nanoflowers	Diabetic wound healing	Controlled release for bacterial elimination, tissue regeneration, and neovascularization	Exosomes release therapeutic agents targeting oxidative wound microenvironment, improving tissue regeneration and healing	[172]
B-cell-derived EVs loaded with zinc oxide nanocrystals (ZnO NCs) and anti-CD20 antibodies	Lymphoma	Target and enhance cytotoxicity against CD20^+^ cancer cells via ultrasound activation	Zinc oxide nanocrystals and anti-CD20 antibodies delivered by EVs are activated by high-energy ultrasound, enhancing cytotoxicity	[173]

#### Engineered EV-nanosystems for multimodal synergistic cancer immunotherapy

Cancer’s complexity, driven by multiple signaling pathways and stages of tumor progression, makes it difficult for any single treatment to completely eradicate tumors or prevent metastasis. As such, multimodal synergistic therapy, which integrates various therapeutic modalities into a unified platform, has emerged as a promising strategy to leverage the strengths of each modality and improve overall treatment outcomes [175]. One of the most effective approaches within this framework is Photodynamic Therapy (PDT), which utilizes photosensitizers, light, and oxygen to generate cytotoxic reactive oxygen species (ROS), leading to tumor cell death. However, the hypoxic microenvironment often found in tumors limits PDT’s effectiveness, as oxygen is a critical component in the process [176]. To overcome this limitation, PDT is frequently combined with chemotherapy, which not only enhances the therapeutic efficacy but also improves oxygen delivery to the tumor [177].

A significant advancement in this area was made by Liu et al., who developed a hybrid liposome-based system incorporating nanoplatinum (nano-Pt) and the photosensitizing agent vetipofen (VP), combined with macrophage cell membranes for biomimetic targeting. This system demonstrated efficient chemo-PDT in mouse models. The nano-Pt acted as a catalyst at the tumor site, producing oxygen and enhancing VP-mediated PDT. Furthermore, nano-Pt also facilitated the release of chemotherapy agents, improving tumor penetration and therapeutic efficacy. The incorporation of macrophage cell membranes helped target the tumor more specifically, further enhancing the overall treatment response [178].

Similarly, in another innovative study, Liu et al. engineered a novel biomimetic nanocarrier using erythrocyte membranes, which encapsulated a combination of bovine serum albumin (BSA), 1,2-diaminocyclohexane-platinum, and indocyanine green (ICG). This erythrocyte membrane nanocarrier exhibited enhanced immune evasion and tumor-targeting properties, which significantly increased circulation time and tumor accumulation. Upon exposure to NIR light, the erythrocyte membrane ruptured, releasing the encapsulated drugs. The platinum compound effectively inhibited cancer cell proliferation through DNA damage, while ICG generated cytotoxic singlet oxygen and heat under NIR, promoting tumor cell apoptosis. This design exemplifies the potential of combining PDT with other therapeutic modalities, such as chemotherapy, in a single platform to overcome the limitations of each individual treatment [179].

Beyond these hybrid nanocarrier systems, another intriguing approach involves utilizing EVs, which are naturally derived and capable of efficient cellular communication. Recent studies have shown the potential of EVs as delivery systems in cancer therapy. For example, a study on osteosarcoma (OS)—a rare and aggressive tumor with a high metastatic rate—demonstrated the potential of EVs modified with folic acid to deliver Hdac1 siRNA and zoledronic acid (zol). This targeted strategy showed enhanced tumor targeting and significant therapeutic effects in OS management, addressing both drug resistance and toxicity challenges associated with traditional therapies [180].

The integration of engineered EVs with multimodal therapeutic strategies, such as PDT, chemotherapy, and gene therapy, represents a powerful approach to overcoming the limitations of individual treatments (Table 6). By incorporating various therapeutic elements into a single nanosystem, these engineered EV-nanosystems are capable of synergistically enhancing tumor-targeted delivery, improving therapeutic efficacy, and ultimately providing a more comprehensive and effective treatment for various cancer types.

**Table 6:** Engineered EV-Nanosystems for Multimodal Synergistic Cancer Immunotherapy

Evs Type	Cancer Type	Biological role	Mechanism	Reference
Hybrid liposome-based system (nano-Pt + VP, hybridized with macrophage cell membranes)	Not specified	Enhance chemo-PDT efficacy	Nano-Pt catalyzes oxygen production at the tumor site, enhancing VP-mediated PDT, and promotes chemotherapy via improved tumor penetration	[178]
Biomimetic nanocarrier (BSA + 1,2-diaminocyclohexane-platinum + ICG, embedded in erythrocyte membrane)	Not specified	Increase circulation time and tumor accumulation, promote tumor cell apoptosis	Erythrocyte membrane rupture under NIR light releases drugs; platinum mediates DNA damage, and ICG produces cytotoxic singlet oxygen and heat	[179]
Extracellular vesicles (EVs) modified with folic acid (delivering Hdac1 siRNA and zoledronic acid)	Osteosarcoma (OS)	Enhance tumor targeting and therapeutic effects	Targeted delivery of Hdac1 siRNA and zoledronic acid to cancer cells, addressing drug resistance and toxicity	[180]

### Challenges in clinical translation

**Fig. 7:**
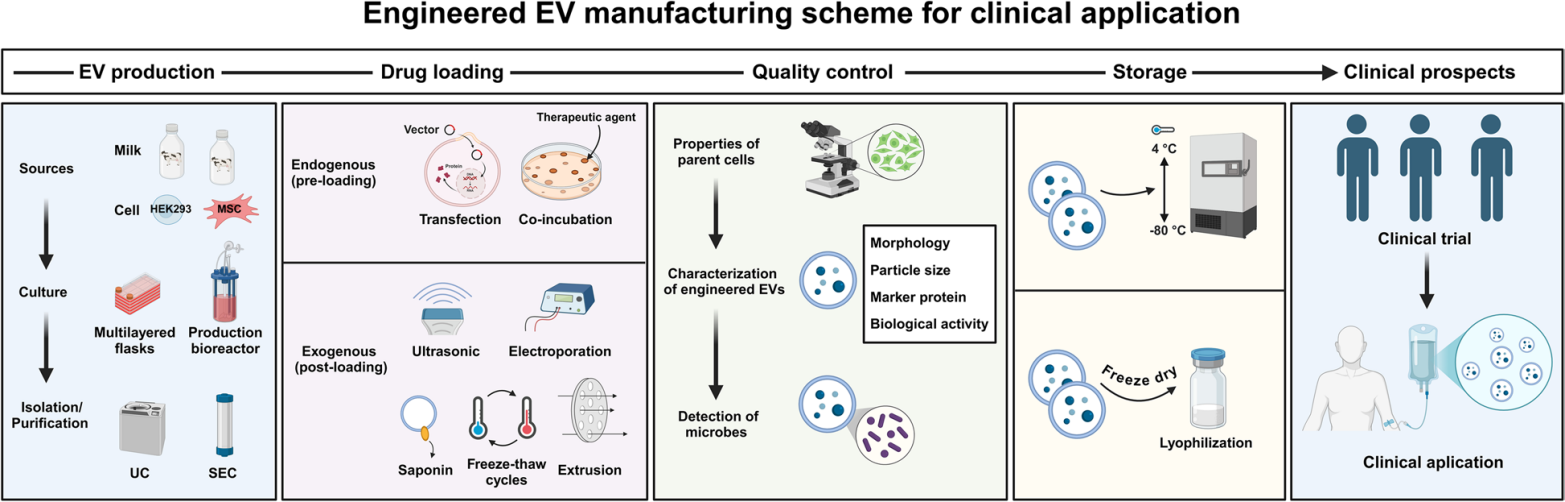
Engineered EV manufacturing scheme for clinical application. The development of engineered EV treatment under GMP conditions starts from the production of EV, whereas the isolation and purification methods still lack a universal approach, and the combination of them, such as UC and SEC, is recommended. Subsequently, the drug loading was conducted by endogenous or exogenous manner dependent on the types of therapeutic agents. However, how to improve the loading efficacy and maintain EV stability remains challenging. After the engineered process, quality control of obtained EVs is strongly suggested to test their uniformity. 4℃ and −80℃ are common used for the storage of EVs, whereas the lyophillization emerges as an alternative method. Finally, before clinical application, clinical trials are required to further evaluate the safety and efficacy of the engineered EVs in patients with cancer. Created with BioRender.com

Despite encouraging results achieved in diverse cell and animal models, the clinical translation of engineered EVs for tumor therapy remains in the preliminary stage. One of the greatest challenges is the development of Good Manufacturing Practice (GMP) standards to produce engineered EVs on a large scale and ensure they meet the rigorous standards of clinical-grade quality (Fig. 7). While EVs are emerging as a promising cell-free immunotherapy strategy, with significant success in laboratory and preclinical studies, raising hopes for their clinical translation in cancer treatment, several obstacles remain in their clinical application.

A major challenge lies in identifying a scalable and safe EV source for therapeutic use. Various strategies have been proposed, including milk-derived EVs, plant-derived EVs, and synthetic exosome-like particles, each with its own set of advantages and limitations. Milk-derived EVs, for example, are non-immunogenic and easy to isolate, but their purity and consistency can vary. Plant-derived EVs are a promising alternative due to their abundance and ease of production, but concerns over potential contaminants or foreign antigens need to be addressed. Synthetic exosome-like particles offer controlled composition and functionality but may lack the natural cargo and biological activity of native EVs. The identification and optimization of such sources are crucial for ensuring the safety, efficacy, and scalability of EV-based therapies in clinical settings.

In addition, the heterogeneity and functional instability of EVs pose significant challenges. Common isolation techniques, such as continuous centrifugation, tangential flow filtration, and ultracentrifugation, struggle to effectively separate EV subgroups, often compromising EV integrity or causing aggregation. While tangential flow filtration produces higher quantities with less aggregation, it still cannot meet the demands for large-scale production. Size-exclusion chromatography, which avoids harsh centrifugal forces and better preserves EV integrity, offers a solution, but it is limited by low yield and high cost. Therefore, developing improved or more efficient methods for preparing clinical-grade EVs is essential.

Another significant challenge is the lack of a clear safety profile for EVs. In-depth studies are needed to better understand their roles and mechanisms in cancer therapy. Tumor-derived exosomes (TEX), in particular, present controversies in research. Some studies suggest that TEX have homologous targeting and immunogenicity, making them promising therapeutic vectors or tumor vaccines. However, other research indicates that TEX can suppress CD8^+^ T cell proliferation by lowering Interleukin-2 (IL-2) levels, or induce apoptosis in CD8^+^ T cells via the FasL-TRAIL pathway or Galectin-9 [181]. Furthermore, TEX can promote immune tolerance, suppress host immune responses, and activate pathways that encourage tumor growth, metastasis, and angiogenesis, thereby helping tumors evade immune surveillance and resist treatment [182]. Balancing the therapeutic efficacy of EVs with their potential side effects remains a critical concern [183].

Additionally, while efforts to engineer the surfaces and cargo of EVs using both chemical and biological methods aim to enhance their beneficial properties and reduce harmful effects, designing EVs that maintain their functionality without compromising their therapeutic potential continues to be a major challenge. As such, a greater understanding of EV biology and the development of improved engineering techniques will be crucial to advancing EV-based therapies in clinical oncology.

### Summary and prospects

**Fig. 8:**
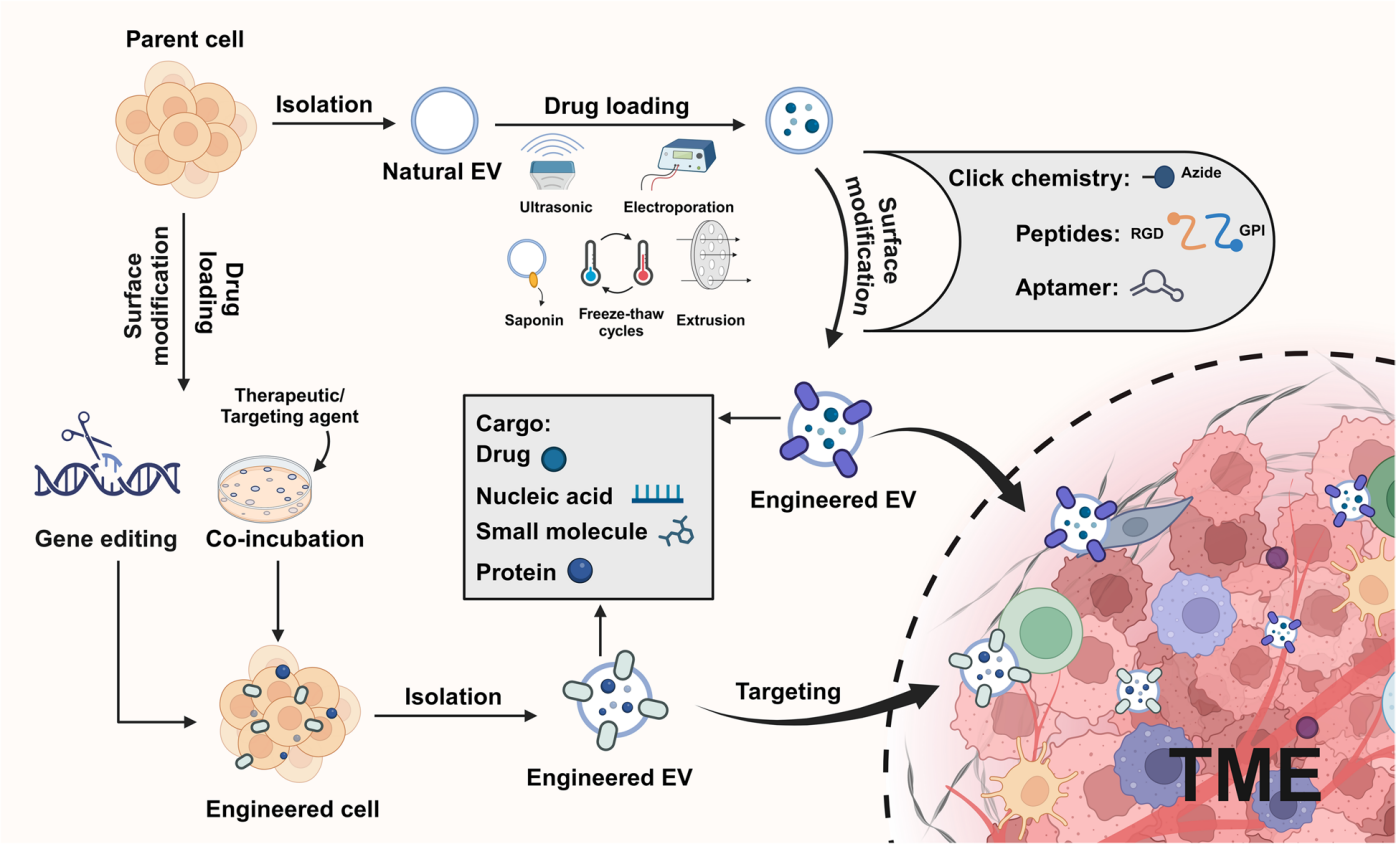
Strategies of engineered EVs for cancer therapy by targeting TME. Engineered EVs targeting the TME can be obtained from engineering parent cell or direct transformation of natural EVs. For parent cells, gene editing and co-incubation methods are the two primary approaches, whereas the drug loading and surface modification approaches of natural EVs are mainly based on the properties of utilized agents. Created with BioRender.com

EVs have rapidly emerged as a promising cell-free immunotherapy platform, demonstrating remarkable potential in both preclinical and clinical studies. These vesicles, including Exos and MVs, play crucial roles in intercellular communication by transferring bioactive molecules such as proteins, lipids, and nucleic acids to target cells. This inherent ability makes EVs highly attractive for cancer immunotherapy, either as vehicles for targeted therapeutic payloads or as cancer vaccines. The growing interest in EVs is evidenced by over ten ongoing clinical studies exploring EV-mediated cancer immunotherapy, reflecting increasing confidence in their clinical application. With the help of advanced engineering techniques, EVs have shown considerable promise in enhancing anti-tumor therapy (Fig. 8).

However, despite these exciting developments, several challenges still hinder the clinical translation of EV-based therapies. One of the primary hurdles is the heterogeneity and instability of EVs, which can lead to inconsistent therapeutic outcomes. Current isolation techniques-such as ultracentrifugation, tangential flow filtration, and size exclusion chromatography-still face limitations. For instance, while tangential flow filtration offers higher yields with less aggregation, it remains inadequate for large-scale production, and size exclusion chromatography, though gentler on EVs, suffers from low yield and high costs. Therefore, the need for more efficient, scalable methods to produce clinical-grade EVs remains critical. Another significant challenge lies in the safety profile of EVs, which has yet to be fully understood. While TEX have shown potential for homologous targeting and immunogenicity, making them suitable as therapeutic vectors or tumor vaccines, conflicting research has raised concerns. For example, TEX may inhibit CD8^+^ T cell proliferation by reducing IL-2 levels or even induce apoptosis in CD8^+^ T cells via pathways like FasL-trail or Galectin-9. Furthermore, TEX could suppress immune responses, promote immune tolerance, and contribute to tumor progression by activating pathways that encourage metastasis, angiogenesis, and immune evasion. These conflicting findings highlight the necessity of carefully balancing the therapeutic benefits of EV-based treatments with their potential risks.

To overcome these limitations, future research must focus on several key areas. One critical direction is the development of advanced methods for isolating and characterizing EVs with high purity, consistency, and specificity. Techniques such as microfluidics, immunoaffinity capture, and innovative chromatography approaches could enable more precise separation of EV subpopulations while maintaining their functional integrity. Additionally, engineering EVs through both chemical and biological modifications can help to enhance their therapeutic properties while minimizing adverse effects. This includes surface modifications for better targeting specificity, precise loading of therapeutic cargo, and strategies to prevent premature immune clearance. Moreover, leveraging gene-editing tools like CRISPR/Cas9 could enable the creation of designer EVs with tailored functionalities, taking precision medicine to new levels. A comprehensive understanding of the safety profile of engineered EVs is essential for their successful clinical application. Long-term studies on their biodistribution, pharmacokinetics, and potential toxicity will be crucial in evaluating their safety. Additionally, investigating the interaction between EVs and the immune system will help refine their use and mitigate adverse effects. To scale up EV-based therapies for clinical use, it is essential to establish standardized and reproducible methods for the large-scale production of clinical-grade EVs. These efforts should focus on developing protocols for EV isolation, purification, storage, and quality control, ensuring consistency across batches and compliance with regulatory standards.Rigorous clinical trials will also be necessary to validate the safety and efficacy of EV-based therapies. Collaborative efforts between academia, industry, and regulatory bodies will be pivotal in developing standardized guidelines and regulatory frameworks that facilitate the clinical use of EVs. Such partnerships can accelerate the translation of EV-based therapies from the lab to clinical practice.

Looking ahead, the future of EVs in clinical applications holds immense promise. Their unique properties, such as biocompatibility, ability to cross biological barriers, and capacity for targeted delivery, make them ideal candidates for precision cancer immunotherapy and other medical applications. By addressing current challenges and deepening our understanding of EV biology and engineering, we can unlock new opportunities for EV-based therapies. Engineered EVs have the potential to revolutionize personalized medicine, offering targeted, efficient, and safe treatment options for various diseases. As research progresses, EV-based therapies are expected to become a mainstay in clinical practice, bringing new hope to patients and advancing the field of cell-free immunotherapy.

## Data Availability

No datasets were generated or analysed during the current study.

## Abbreviations

Abs: Apoptotic bodies
BM-MSCs: Bone marrow mesenchymal stem cells
CD47: CD47-binding peptides
Cis: Checkpoint inhibitors
CAR: Chimeric antigen receptor
circRNA: Circular RNA
CRS: Cytokine release syndrome
CTLs: Cytotoxic T lymphocytes
DCs: Dendritic cells
DD: Drug delivery
Ectos: Ectosomes
EGFR: Epidermal growth factor receptor
EpCAM: Epithelial cell adhesion molecule
Exo-vaccine: Exosomal vaccine
Exos: Exosomes
EVs: Extracellular vesicles
GETs: Gene-editing tools
GE-Exosomes: Gene-engineered exosomes
Gal-9: Galectin-9
HCC: Hepatocellular carcinoma
HER2^+^ BC: Her2-positive breast cancer
HNPs: Hybrid nanoparticles
HIPEC: Hyperthermic intraperitoneal chemotherapy
IL-2: Interleukin-2
IL-6: Interleukin-6
LAMP-2B: Lysosomal-associated membrane protein 2B
lncRNA: Long non-coding RNA
MF: Magnetic fields
MHC: Major histocompatibility complex
MSCs: Mesenchymal stem cells
miRNA: MicroRNA
MVs: Microvesicles
M1-Exos: M1 macrophage-derived exosomes
MSLN: Mesothelin
NK: Natural killer
NIR: Near-infrared
OTE: Off-target effects
OX40L: OX40 ligand
PDT: Photodynamic therapy
PTT: Photothermal therapy
PD-1: Programmed cell death protein 1
PDGFR: Platelet-derived growth factor receptor
Tregs: Regulatory T cells
rDNA: Recombinant DNA technology
SEC: Size-exclusion chromatography
siRNA: Small interfering RNA
STING: Stimulator of interferon genes
Ser/Thr kinase: Serine/threonine protein kinase
TE: Therapeutic effectiveness
TSL: Thermosensitive liposomes
TAAs: Tumor-associated antigens
TAMs: Tumor-associated macrophages
TEX: Tumor-derived extracellular vesicles
TIE: Tumor immune evasion
TME: Tumor microenvironment
IFN: Type I interferon
Wnt: Wnt signaling pathway
BPQDs: Black phosphorus quantum dots
G-EXOs: Glioma-derived exosomes
GENPs: Golgi apparatus-PD-L1-/- exosome hybrid membrane-coated nanoparticles
DT-Exo-STING: DC-tumor hybrid cell-derived chimeric exosome loaded with STING agonists
HNSCC: Head and neck squamous cell carcinoma
Hy-M-Exo: Hybrid nanovaccine
NK-NPs: NK cell membrane
TNBC: Triple-negative breast cancer
PTX: Paclitaxel
TIME: Tumor immune microenvironment
ICIs: Immune checkpoint inhibitors
M1 NVs: M1-type nanovesicles
CDNs: Cyclic dinucleotides
PDAC: Pancreatic ductal adenocarcinoma
NPC: Nasopharyngeal carcinoma
LMP1: Latent membrane protein 1
scFvs: Single chain variable fragments
RT-MPs: RT-induced microparticles
ICB: Immune checkpoint blockade
OMVs: Outer membrane vesicles
CTLs: Cytotoxic T lymphocytes
T-EXOs: Tumor-derived exosomes
NSCLC: Non-small-cell lung cancer
MGS: Mesoporous gold sensor
BsEVs: Bispecific EVs
PAMPs: Pathogen-associated molecular patterns
MPC: Peritoneal carcinomatosis
hGLVs: Hybrid therapeutic nanovesicles
BBB: Blood-brain barrier
GBM: Glioblastoma
TNH: TrojanNanoHorse
ROS: Reactive oxygen species
VP: Vetipofen
nano-Pt: Nanoplatinum
BSA: Bovine serum albumin
ICG: Indocyanine green
OS: Osteosarcoma
zol: Zoledronic acid
